# Slx5p‐Slx8p Promotes Accurate Chromosome Segregation by Mediating the Degradation of Synaptonemal Complex Components during Meiosis

**DOI:** 10.1002/advs.201900739

**Published:** 2020-01-01

**Authors:** Chao Liu, Haichao Zhao, Sai Xiao, Tingting Han, Yinghong Chen, Tong Wang, Yanjie Ma, Hui Gao, Zhiping Xie, Li‐Lin Du, Jian Li, Guoping Li, Wei Li

**Affiliations:** ^1^ State Key Laboratory of Stem Cell and Reproductive Biology Institute of Zoology Chinese Academy of Sciences Beijing 100101 P. R. China; ^2^ College of Life Sciences University of Chinese Academy of Sciences Beijing 100049 P. R. China; ^3^ The Key Laboratory of Geriatrics Beijing Institute of Geriatrics Beijing Hospital National Center of Gerontology National Health Commission Institute of Geriatric Medicine Chinese Academy of Medical Sciences Beijing 100730 P. R. China; ^4^ Joint International Research Laboratory of Metabolic & Developmental Sciences School of Life Sciences and Biotechnology Shanghai Jiao Tong University Shanghai 200240 P. R. China; ^5^ National Institute of Biological Sciences Beijing 102206 P. R. China

**Keywords:** chromosome segregation, meiosis, Slx5p‐Slx8p, small ubiquitin‐like modifier (SUMO)‐ubiquitination crosstalk, synaptonemal complexes

## Abstract

Meiosis increases genetic diversity, yet the genome complement needs to be stable to ensure offspring viability. Both small ubiquitin‐like modifier (SUMO) and ubiquitin have been reported to participate in meiotic regulation, yet functions of the SUMO‐ubiquitination crosstalk in meiosis remain unclear. Here, it is reported that a SUMO‐targeted ubiquitin ligase, Slx8p, promotes accurate chromosome segregation during meiosis, since the deletion of *SLX8* leads to increased aneuploidy due to a defect in synaptonemal complex (SC) component degradation. Both the RING domain and SUMO interacting motifs of Slx8p are essential for meiotic progression and maintaining spore viability, and the expression of tetraubiquitin fused with SUMO partially rescues meiotic defects in the *SLX8*‐deletion strain. Furthermore, Slx5p‐Slx8p can directly add ubiquitin to SUMOylated Zip1p and Ecm11p, and forced degradation of Ecm11p partially rescues the sporulation defects of the *SLX8* deletion strain. These findings provide a mechanism for SC disassembly and reveal that the crosstalk between SUMOylation and ubiquitination facilitates accurate chromosome segregation by promoting SC component degradation during meiosis.

## Graphical abstract

As a heterodimeric small ubiquitin‐like modifier (SUMO) targeted ubiquitin ligase (STUbL), Slx5p‐Slx8p can recognize and ubiquitinate SUMOylated synaptonemal complex components to promote their degradation, thus ensuring accurate chromosome segregation during meiosis.

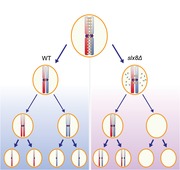

## 1 Introduction

Meiosis is a fundamental process of sexual reproduction in eukaryotes, which halves the genome by a single round of DNA replication followed by two consecutive cell divisions: meiosis I (MI) and meiosis II (MII).^1^ During meiosis, homologous chromosome segregate to opposite spindle poles during MI, while sister chromatids remain associated until their segregation during MII. A remarkable feature of meiosis is a prolonged prophase I: a series of events take place during this stage to ensure accurate homologous chromosome segregation during MI, including homologous chromosome recognition, pairing and synapsis, the generation of programmed DNA double‐strand breaks (DSBs), homologous recombination, and late prophase I chromosome remodeling.^2^, ^3^ Inaccuracy in these events can cause genome instability and errors in meiotic chromosome segregation, as well as the production of aneuploidy, which is associated with infertility, embryonic lethality, or pronounced developmental defects.^4^, ^5^

A zipper‐like tripartite proteinaceous structure, known as the synaptonemal complex (SC), is a hallmark of prophase I, and it is required for both homologous recombination and stabilizing the interactions between homologous chromosomes to ensure accurate chromosome segregation.^3^, ^6^, ^7^ The SC is composed of the following: the lateral elements (LEs) that assemble and run down the length of meiotic chromosome axes in two parallel tracks, a series of transverse filaments (TFs) that span the region between the two homologs and bridge the parallel homologous axes; and the central element (CE) proteins that are located along the center of the SC to stabilize the complex.^6^, ^7^ Several components of the SC have been identified: Red1p, Hop1p, and Mek1p are parts of LEs; Zip1p exists in TFs; Ecm11p, Gmc2p exist in CE in *Saccharomyces cerevisiae*.^3^, ^7^ Although the components of the SC share little conservation at the amino acid sequence level among different organisms, the ultrastructure and biological functions of the SC are similar to each other in most eukaryotes.^7^, ^8^, ^9^

The SC dynamically changes during MI and goes through three stages that include assembly, a highly dynamic steady state, and disassembly.^3^ In budding yeast, SC assembly begins with the recruitment of Zip3p and Zip1p at the sites of meiotic recombination events.^10^, ^11^ These events are followed by the interaction of SUMOylated Red1p with Zip1p and further recruitment of Zip2p, Zip4p, and Spo16p to form the synapsis initiation complex (SIC).^12^, ^13^ The maturation of the SC is regulated by a positive feedback system: Zip1p initiates the SUMOylation of Ecm11p; poly‐SUMOylated Ecm11p promotes more Zip1p assembly; and then more Zip1p facilitates further loading of Ecm11p/Gmc2p and their SUMOylated forms.^14^ At the pachytene stage, the SC is assembled at the interface of nearly all lengthwise‐aligned homologous chromosome pairs.^15^ Upon pachytene exit, the SC starts to disassemble in an asymmetric manner and SC proteins are retained at specific chromosome subdomains until late prophase I.^16^, ^17^ Compared with SC assembly, the mechanism underlying SC disassembly is not well understood.

During meiosis, both SC formation and disassembly are essential for proper segregation of homologs.^2^, ^7^, ^18^ The SC formation can stabilize the chromosome structure and sense the presence of aberrantly entangled chromosomes.^19^ Once the SC is disassembled, crossovers tether the homologs to each other and orient them for segregation.^2^ In addition to mediating the close apposition of homologous chromosomes during mid‐meiotic prophase, some SC components participate in centromere coupling and pairing. In the leptotene, Zip1 first appears as dispersed punctate foci at or near the centromeres, and mediates the homology‐independent association of centromeres.^20^, ^21^ When the SC disassembles in late prophase, Zip1p remains at the paired centromeres, leaving the homologous partners joined by only chiasmata and centromere pairing.^22^ Zip3p is also important for nonexchange chromosome segregation, and it was shown that *ZIP3* deletion impairs both the tethering of nonexchange chromosomes and the localization of Zip1p to centromeres.^22^ The disassembly of the SC at paired centromeres could allow for chromosome segregation at meiosis I.^7^

Post‐translational modifications (PTMs) of SC‐related proteins, such as SUMOylation, phosphorylation, and N‐terminal acetylation, have emerged as important regulators of SC dynamics.^3^, ^7^ Recent studies have reported that SUMO, ubiquitin, and proteasomes coordinately work to regulate the key events of meiotic prophase I.^23^, ^24^ RNF212 and HEI10 define an axis‐associated, SUMO‐ubiquitin‐proteasome relay that may mediate turnover of a subset of recombination factors that act after DMC1‐promoted homolog pairing.^23^ SUMO E3 ligase Zip3p and SC protein Zip1p are required for proteasome particle recruitment to chromosomes during meiosis, which is essential for SC assembly and crossover formation.^24^ However, the functional role and molecular mechanism underlying the SUMO‐ubiquitin‐proteasome system in prophase I remains poorly understood, and whether a mediator exists to conduct these PTM signals in prophase I still requires further investigation.

The SUMO‐targeted ubiquitin ligase (STUbL) is defined as a specific class of ubiquitin ligases (E3) that recognize the SUMOylated substrates via SUMO‐interaction motifs and ubiquitinate them via the RING domain, and this process is indispensable for the crosstalk between SUMOylation and ubiquitination.^25^ To delineate the functional roles of SUMO‐ubiquitin crosstalk during meiosis, we tested all the potential functions of the STUbLs during yeast meiosis. We found that only the disruption of Slx5p‐Slx8p significantly influenced the meiotic progression in yeast, where it mainly participated in the degradation of SC components. The function of Slx8p during meiosis is dependent on its STUbL activity, and we identified Ecm11p as the key substrate. These findings reveal that the crosstalk between SUMOylation and ubiquitination promotes proper chromosomal segregation by mediating the degradation of SC components during meiosis.

## 2 Results

### 2.1 Slx5p‐Slx8p Is the Key STUbL that Regulates Meiosis

**Figure 1:**
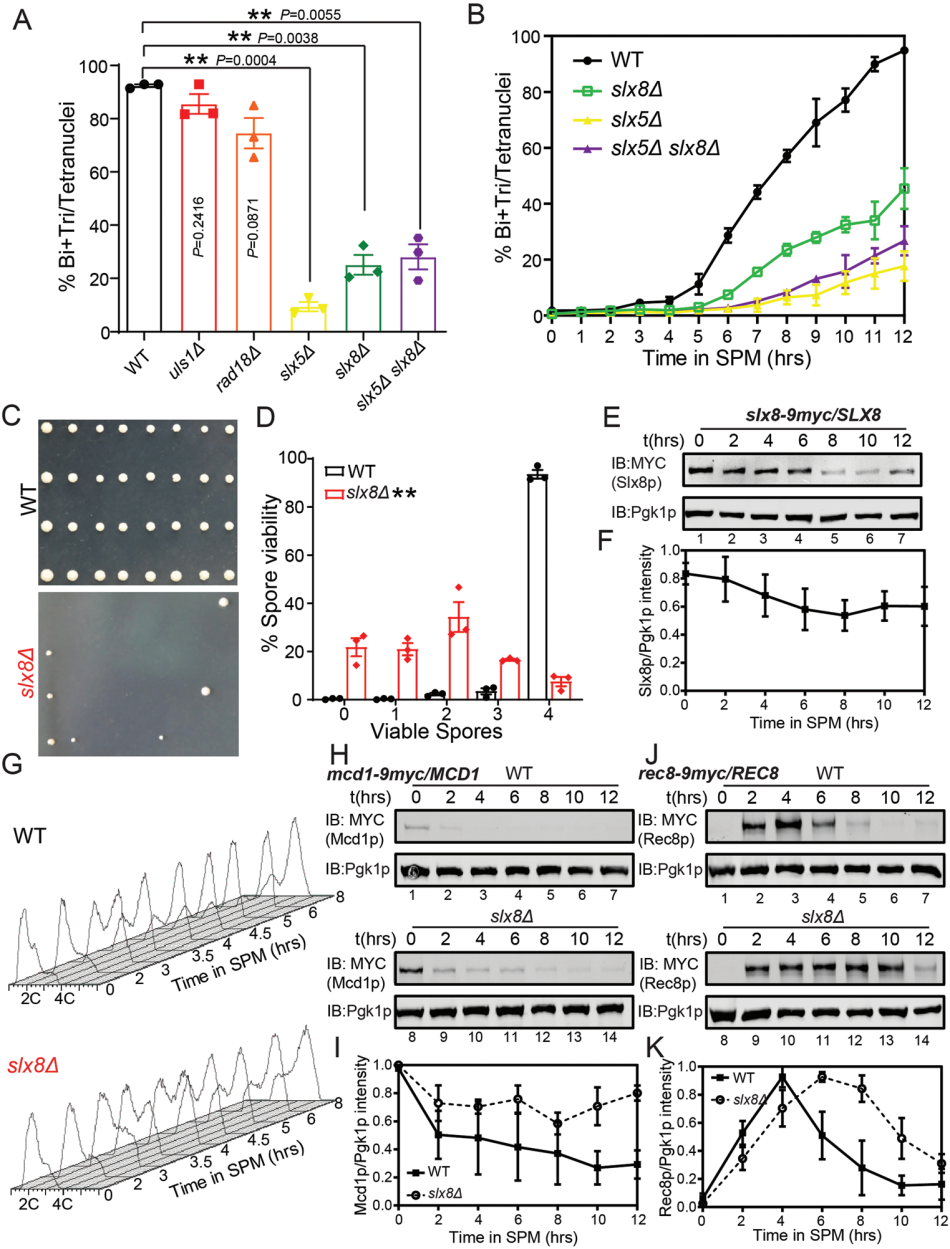
Slx5p‐Slx8p is the key STUbL to regulate meiosis. A) Functional screening of STUbL genes during yeast meiosis. WT and mutant strains were induced to sporulate and analyzed after 24 h. The divided nuclei were stained with DAPI, and more than 400 nuclei were scored for each experiment (*n* = 3 independent experiments). Data are presented as mean ± SEM. **P < 0.01. Two‐sided *t*‐tests were used for statistical analyses. B) The disruption of *SLX5* and *SLX8* impaired meiotic progression. WT, *slx5Δ*, *slx8Δ*, and *slx5Δ slx8Δ* cells were induced to undergo meiosis by transfer to SPM and analyzed at different time points, with more than 180 nuclei scored for each time point (*n* = 3 independent experiments). Data are presented as mean ± SEM. C) The spore viability of WT and *slx8Δ* cells. Tetrad analysis of WT and *slx8Δ* diploids was performed by dissection of tetrads after sporulation. D) Quantification of spore viability in WT and *slx8Δ* cells. More than 200 tetrads were scored for each time point (*n* = 3 independent experiments). Data are presented as mean ± SEM. Two‐sided *t*‐tests and *G*‐tests were used for statistical analyses. The asterisks indicate statistically significantly improved viability frequency of *slx8Δ* cells compared to WT cells (*P* < 0.0001, *G*‐test for homogeneity). *P* = 0.0014 proportion of four‐viable spores (*t*‐test, WT vs *slx8Δ*). E) Slx8p was predominantly expressed at the early stage of meiosis. The WT strain expressing Slx8p‐9MYC in one allele was incubated in SPM, and samples were collected at different time points after sporulation induction. Expression of Slx8p‐9MYC was analyzed over time by immunoblotting using an anti‐MYC antibody. Pgk1p served as a loading control. F) Ratios of Slx8p/Pgk1p levels were normalized to the maximum Slx8p/Pgk1p ratio (*n* = 3 independent experiments). Data are presented as mean ± SEM. G) Premeiotic DNA replication was delayed in the *slx8Δ* strain during sporulation. H–K) The expression of the mitotic and meiotic cohesin subunits in the *slx8Δ* cells. Expression of H) Mcd1p and J) Rec8p were detected in WT and *slx8Δ* cells during sporulation by immunoblotting using the anti‐MYC antibody. Pgk1p served as a loading control. Ratios of I) Mcd1p/Pgk1p levels were normalized to the maximum Mcd1p/Pgk1p ratio (*n* = 3 independent experiments). Ratios of K) Rec8p/Pgk1p levels were normalized to the maximum Rec8p/Pgk1p ratio (*n* = 3 independent experiments). Data are presented as mean ± SEM. See also Figures S1 and S2 in the Supporting Information.

Three STUbLs have been reported to play roles in budding yeast, including Uls1p, Rad18p, and Slx5p‐Slx8p.^25^ To study the functional roles of the STUbLs during meiosis, we tested all STUbL enzymes by deleting *ULS1*, *RAD18*, *SLX5*, and *SLX8* in SK1 background *S. cerevisiae*. After incubating in sporulation medium for 24 h, we found *SLX5*‐ and *SLX8*‐deleted cells showed obvious impairment in the sporulation process (**Figure** **1**A). Furthermore, the double deletion of *SLX5* and *SLX8* also influenced the meiotic progression compared to the wild‐type (WT) strain (Figure 1A), suggesting that Slx5p‐Slx8p was the key STUbL responsible for regulating yeast sporulation. To further confirm our finding, we evaluated the meiotic progression in WT, *slx5Δ*, *slx8Δ*, and *slx5Δ slx8Δ* strains, and found that sporulation in *slx5Δ*, *slx8Δ*, and *slx5Δ slx8Δ* strains showed a delay at the early stages of meiosis (Figure 1B). Therefore, Slx5p‐Slx8p is the key STUbL for the regulation of meiosis, and the disruption of Slx5p‐Slx8p obviously impairs meiotic progression. As it was difficult to detect the spore formation in *slx5Δ* and *slx5Δ slx8Δ* cells after incubating in sporulation medium for 24 h (Figure S1A, Supporting Information), we examined the spore viability in *slx8Δ* cells and found most of the tetrad‐derived spores in the *slx8Δ* mutant cells were inviable (Figure 1C,D). This result indicates Slx8p should be required for proper chromosome segregation during meiosis, and we selected Slx8p for further investigation.

### 2.2 The Disruption of *SLX8* Impairs Meiotic Cell Division

To investigate the physiological function of Slx8p, we evaluated its expression during yeast meiosis. We found that the protein was predominantly expressed at the early stages of meiosis (Figure 1E,F), indicating Slx8p might regulate early events of meiosis. To identify meiotic processes controlled by Slx8p, we first compared premeiotic DNA synthesis in the *slx8Δ* strain with that in WT cells using flow cytometry analysis. Although the premeiotic DNA replication showed a minor delay during the early stage of sporulation after *SLX8* depletion, DNA replication was completed after 6 h in sporulation medium in both WT and *slx8Δ* cells (Figure 1G; Figures S1B and S2, Supporting Information). This result suggests that the disruption of *SLX8* has a modest effect on premeiotic DNA replication. Accompanying premeiotic DNA replication, the meiosis‐specific cohesin subunit, Rec8p, is known to replace the mitotic cohesin subunit Mcd1p to form the predominant form of cohesin during meiosis.^26^ We next observed the expression of the cohesin subunit during meiosis and found Mcd1p degradation and Rec8p expression were both somewhat delayed in *slx8Δ* cells (Figure 1H–J). Furthermore, the degradation of Rec8p was obviously delayed in *slx8Δ* cells (Figure 1J,K). Taken together, the *SLX8* deletion delays premeiotic DNA replication and meiotic cohesin expression, and impairs cell divisions during meiosis.

### 2.3 *SLX8* Deletion Leads to Extended Prophase I and Metaphase I

**Figure 2:**
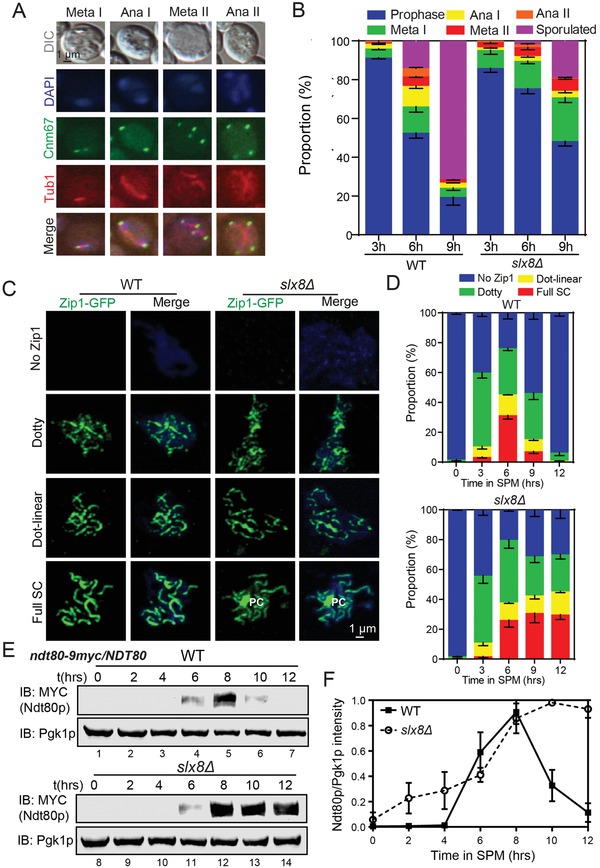
The disruption of Slx8p causes extended prophase I and metaphase I. A) Characterization of meiotic cell cycle by SPB and spindle. *CNM67*‐GFP (green)/mCherry‐*TUB1* (red) cells were sporulated in SPM and samples were collected at indicated time points, and nuclei were stained with DAPI (blue). B) The proportion of WT and *slx8Δ* cells at different meiotic stages. More than 180 cells were scored for each time point (*n* = 3 independent experiments). Values of *P* < 0.0001 prophase I at 6 h (WT vs *slx8Δ*), *P* < 0.0001 prophase I at 9 h (WT vs *slx8Δ*), and *P* < 0.0001 metaphase I at 9 h (WT vs *slx8Δ*) were observed. Data are presented as mean ± SEM. Chi‐square tests were used for statistical analyses. C) Characterization of prophase I by morphology of SC (ZIP1‐GFP). WT or *slx8Δ* cells transfected with *pZIP1‐GFP^700^* were incubated in SPM, and harvested at 6 h, and meiotic chromosomes were spread for immunofluorescence microscopy, and nuclei were stained with DAPI (blue). D) The proportion of WT and *slx8Δ* cells at different prophase I stages. More than 200 nuclei were scored for each time point (*n* = 3 independent experiments). Values of *P* < 0.0001 dotty Zip1 at 12 h (WT vs *slx8Δ*), *P* < 0.0001 dot‐liner Zip1 at 12 h (WT vs *slx8Δ*), and *P* < 0.0001 full SC at 12 h (WT vs *slx8Δ*) were observed. Data are presented as mean ± SEM. Chi‐square tests were used for statistical analyses. E) Expression of Ndt80p was detected in WT and *slx8Δ* cells by immunoblotting during sporulation. WT or *slx8Δ* cells expressing the *NDT80*‐9 × MYC in one allele were incubated in SPM, and samples were collected at different time points. Pgk1p served as a loading control. F) Ratios of Ndt80p/Pgk1p levels were normalized to the maximum Ndt80p/Pgk1p ratio (*n* = 3 independent experiments). Data are presented as mean ± SEM. See also Figure S3 in the Supporting Information.

To further determine the precise phases of meiosis that were affected in *slx8Δ* cells, we examined the nuclear division and spindle formation in WT and *slx8Δ* cells by analyzing the states of the spindle and the spindle pole body (SPB) using mCherry‐tubulin and *CNM67*‐GFP (**Figure** **2**A). At 3 h postincubation, we found ≈80% of the WT and *slx8Δ* cells were in prophase I (Figure 2A,B). At 6 h, ≈50% of WT cells had exited prophase I, whereas more than 70% of *slx8Δ* cells were arrested in prophase I (Figure 2A,B). Even at 9 h, more than 40% of *slx8Δ* cells remained arrested in prophase I (Figure 2A,B). In addition, the proportion of cells in metaphase I was also significantly increased in *slx8Δ* cells at 9 h (Figure 2A,B). These results suggest that the disruption of Slx8p extends the period of meiotic prophase I and metaphase I, and Slx8p might play multiple functional roles during meiosis.

### 2.4 Slx8p Is Required for SC Component Degradation

Because meiotic prophase I can be further divided into leptotene, zygotene, pachytene, and diplotene stages as defined by the state of the SC,^27^ we characterized the various stages using Zip1p staining at different time points during meiosis. At 3 h, the foci of Zip1p (leptotene) and dotty linear structures of Zip1p (zygotene) appeared in both WT and *slx8Δ* cells. This was followed by full linear Zip1p staining (pachytene) observed from 6 h (Figure 2C,D). This result indicates the SC assembly occurred similarly in WT and *slx8Δ* cells. In the WT cells, the proportion of nuclei with entirely linear Zip1p decreased from 6 to 12 h, whereas the full linear SC was observed for an extended period in the absence of *SLX8* (Figure 2C,D), indicating that the pachytene period was extended in *slx8Δ* cells. To further validate our findings, we examined the expression of Ndt80p, which is an essential transcription factor for the exit from the pachytene stage.^28^ In WT cells, Ndt80p was highly expressed at 6 h and decreased during sporulation (Figure 2E,F), whereas the transcription factor was highly expressed from 8 to 12 h in *slx8Δ* cells (Figure 2E,F), suggesting that *SLX8* deletion stalls the pachytene exiting.

**Figure 3:**
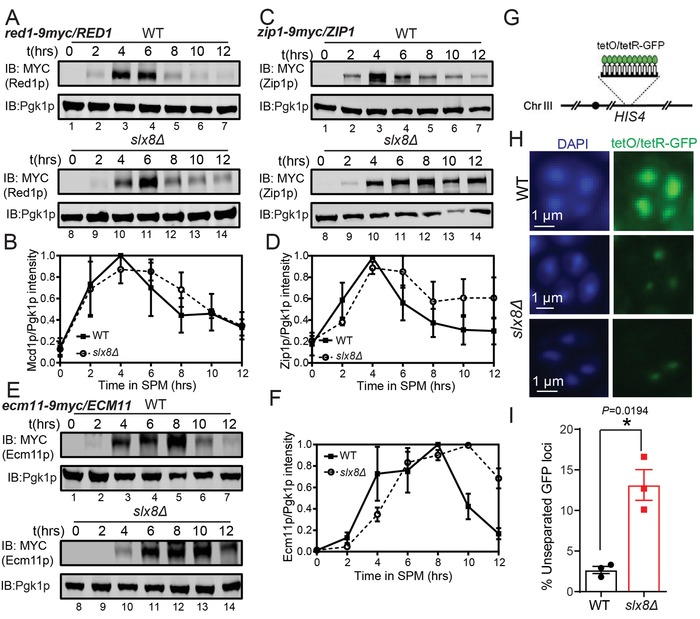
Slx8p is required for accurate chromosome segregation. A–F) The degradation of Zip1p and Ecm11p, but not Red1p, was delayed in *slx8Δ* cells. Expression of A) Red1p, C) Zip1, and E) Ecm11p was detected in WT and *slx8Δ* cells by immunoblotting during sporulation. Pgk1p served as a loading control. Ratios of B) Red1p/Pgk1p, D) Zip1p/Pgk1p, and F) Ecm11p/Pgk1p levels were normalized to the maximum Red1/Pgk1p, Zip1p/Pgk1p, and Ecm11p/Pgk1p ratios (*n* = 3 independent experiments). Data are presented as mean ± SEM. G) Schematic representation of TetO/TetR‐GFP system on Chr III. To label chromosomal tetO repeats with GFP, *pTetR*‐GFP containing a fusion of TetR to GFP under the control of the *URA3* promoter was integrated at *LEU2*, and TetO arrays were integrated at *HIS4* on chromosome III. E) Characterization of homologous chromosome separation. Accurate segregation of chromosome III is indicated by GFP signals in all four nuclei of a cell that has completed meiosis II. Nuclei were stained with DAPI (blue). F) Proportion of meiosis II cells exhibiting chromosome III missegregation. More than 180 cells were scored for each time point (*n* = 3 independent experiments). Data was presented as mean ± SEM. *P < 0.05. Two‐sided *t*‐tests were used for statistical analyses. See also Figures S4 and S5 in the Supporting Information.

The extended persistence of the mature SC could be indicative of impaired meiotic recombination or delayed SC disassembly.^29^, ^30^, ^31^ To distinguish between these two possibilities, we followed the degradation of SC components, including Red1p (LE), Zip1p (TF), and Ecm11p (CE), and the meiotic nuclear divisions. We found no obvious differences in the degradation of Red1p in *slx8Δ* cells compared with WT cells during meiosis (**Figure** **3**A,B). However, some Zip1p protein was retained from 8 to 12 h, even in some proportion of cells that had finished at least one meiotic nuclear division at the same time of *slx8Δ* cells (Figures 1B and 3C,D), indicating that the Zip1p degradation may be impaired in *slx8Δ* cells. Similar degradation defects could also be detected in Ecm11p (Figure 3E,F). It has been reported that the Zip1p polycomplexes (PCs) are frequently juxtaposed with duplicated SPBs,^31^ thus Zip1p PC accumulation suggests a SC component degradation defect in *slx8Δ* cells (Figure S3, Supporting Information).

### 2.5 Slx8p Is Required for Accurate Chromosome Segregation during Meiosis

To exclude the effect on meiotic recombination in *slx8Δ* cells, we engineered *spo11Δ* and *spo11Δ slx8Δ* strains to block meiotic DSB and SC formation.^32^ We found the spore viability in *spo11Δ slx8Δ* cells was similar to that in *spo11Δ* cells (Figure S4B,C, Supporting Information), yet deletion of *SPO11* could not rescue the sporulation defect in *slx8Δ* cells (Figure S4A, Supporting Information). This result suggests the meiosis delay conferred by the *SLX8* deletion was not exclusively linked to meiotic recombination. It has been reported that the SC formation is severely defective in *spo11∆* cells,^33^ and Zip1p remains at the centromeres in *spo11Δ* cells to couple chromosome centromeres.^21^ Absence of SC assembly due to the *SPO11* deletion did not rescue the *slx8Δ* sporulation defect, indicating that the defect of SC disassembly alone on the chromosome arm region is not responsible for the *slx8Δ* sporulation defect (Figure S4A, Supporting Information). At the same time, the dotty Zip1p signal was dramatically increased in *spo11Δ slx8Δ* cells compared to *spo11Δ* cells (Figure S4D, Supporting Information). In *slx8Δ* cells, the proportion of dotty Zip1p signal and Zip1p PCs were significantly higher at 12 h than in the control groups (Figure 2D; Figure S3, Supporting Information), and the proportion of Zip1p signal in the separated nuclei during meiotic division was increased after the deletion of *SLX8* (Figure S5, Supporting Information). These results indicate Slx8p may modulate SC component degradation along chromosome arms, centromeres, or even in regions not associated with chromatin and thereby facilitating meiotic divisions. As it has been proposed that defective SC disassembly might lead to homologous chromosome missegregation,^3^, ^7^ we next investigated whether Slx8p was required for proper chromosome segregation during meiosis using the TetO‐TetR system. TetO arrays were integrated at *HIS4* on chromosome III, and chromosome segregation was examined in the presence of TetR‐GFP^34^ (Figure 3G). We detected more than 10% mis‐segregated chromosomes in *SLX8*‐deleted cells during meiosis, but no more than 3% in WT cells (Figure 3H,I). Our findings indicate that Slx8p is required for accurate chromosome segregation during meiosis.

### 2.6 Slx8p's Role in Meiosis Is Dependent on Its STUbL Activity

**Figure 4:**
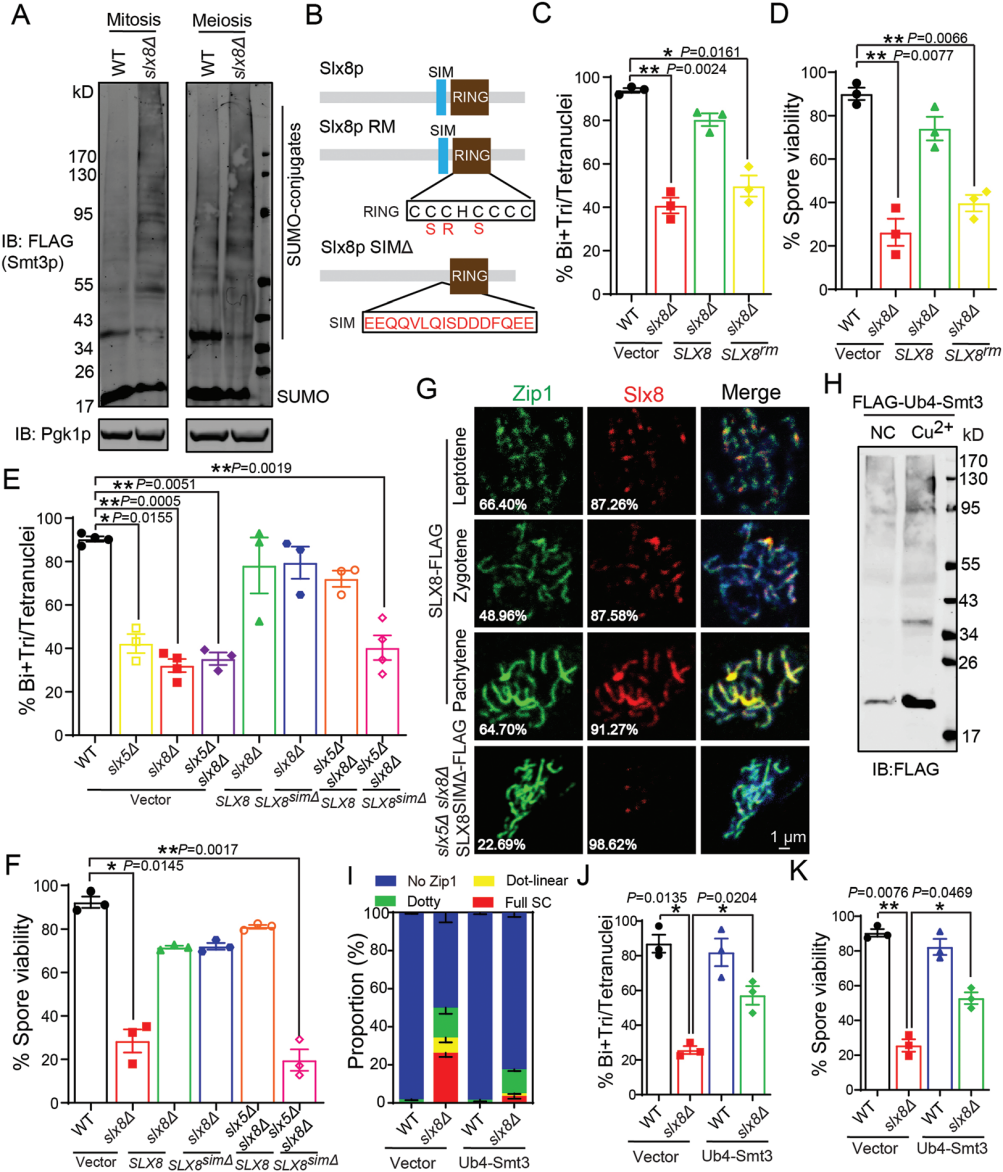
Slx8p facilitates meiotic progression and is dependent on its STUbL activity. A) The disruption of *SLX8* leads to SUMOylated protein accumulation in both mitotic and meiotic cells. Expression of FLAG‐Smt3p was analyzed by immunoblotting using an anti‐FLAG antibody. Pgk1p served as a loading control. B) Schematic representation of the domains of Slx8p, including Slx8p, Slx8p RING mutant (RM), and Slx8p*^simΔ^*. C,D) Slx8p participating in yeast meiosis is dependent on its E3 activity. The proportion of C) divided nuclei and D) spore viability in WT carrying *pRS313*, *slx8Δ* carrying *pRS313*, *slx8Δ* carrying *pRS313‐SLX8*, and *slx8Δ* carrying *pRS313‐SLX8^rm^* cells. More than 150 nuclei and spores were scored for each experiment (*n* = 3 independent experiments). Data are presented as mean ± SEM. *P < 0.05. **P < 0.01. Two‐sided *t*‐tests were used for statistical analyses. E,F) Slx8p participating in yeast meiosis is dependent on its SUMO binding activity. The proportion of E) divided nuclei and F) spore viability in WT carrying *pRS313*, *slx5Δ* carrying *pRS313*, *slx8Δ* carrying *pRS313*, and *slx5Δ slx8Δ* carrying *pRS313*, *slx8Δ* carrying *pRS313‐SLX8* and *slx8Δ* carrying *pRS313‐SLX8^simΔ^* cells, *slx5Δ slx8Δ* carrying *pRS313‐SLX8*, and *slx5Δ slx8Δ* carrying *pRS313‐SLX8^simΔ^* cells. More than 150 nuclei and spores were scored for each experiment (*n* = 3 independent experiments). Data are presented as mean ± SEM. *P < 0.05. **P < 0.01. Two‐sided *t*‐tests were used for statistical analyses. G) Slx8p loads onto meiotic chromosomes during prophase I. WT carrying *pZIP1‐GFP^700^* and *pRS313‐SLX8‐FLAG*, and *slx5Δ slx8Δ* carrying *pZIP1‐GFP^700^* and *pRS313‐SLX8^simΔ^‐FLAG* cells were incubated in SPM and harvested at 6 h, and meiotic chromosomes were spread for immunofluorescence. Nuclei were stained with DAPI (blue). The pixel overlaps of Zip1p and Slx8p were quantified using the IMARIS software. H) The artificial Ub4‐SUMO hybrid could be detected in yeast. Immunoblotting of FLAG was performed in WT carrying *pYEP‐CUP1‐UB4‐SMT3* cells by adding CuSO_4_ (Cu^2+^) or not (NC). I) Proportion of WT and *slx8Δ* cells carrying *pYEP‐CUP1* or *pYEP‐CUP1‐UB4‐SMT3* at the indicated stages of synapsis at 10 h in sporulation medium. More than 200 nuclei were scored for each time point (*n* = 3 independent experiments). *P* < 0.0001 for full SC at 10 h (WT vs *slx8Δ*). Data are presented as mean ± SEM. Chi‐square tests were used for statistical analyses. J,K) Slx8p mediated SUMO‐ubiquitination crosstalk is essential for yeast meiosis. The proportion of J) divided nuclei and K) spore viability in WT, *slx8Δ*, WT Ub4‐Smt3, and *slx8Δ* Ub4‐Smt3 cells. More than 200 nuclei and spores were scored for each experiment (*n* = 3 independent experiments). Data are presented as mean ± SEM. *P < 0.05. **P < 0.01. Two‐sided *t*‐tests were used for statistical analyses. See also Figure S6 in the Supporting Information.

It has been reported that mutations in *SLX8* result in slow growth and hypersensitivity to hydroxyurea due to the accumulation of SUMOylated proteins during mitosis.^35^, ^36^ Thus, we first characterized the SUMO and SUMOylated protein levels in *slx8Δ* cells during meiosis. Both in mitosis and meiosis, *SLX8* deletion led to the accumulation of SUMOylated proteins and a reduction of SUMO monomers (**Figure** **4**A). Based on findings that showed growth defects caused by *slx8Δ* could be rescued by the deletion of SUMO ligase *SIZ1* in mitosis,^36^ we tested whether the deletion of SUMO ligase could rescue the sporulation defect in *slx8Δ* cells. By performing a small‐scale screening of double or triple SUMO ligase mutants, we found that the sporulation efficiency of the mutants showed no significant differences compared with *slx8Δ* cells (Figure S6, Supporting Information), suggesting the functional role of Slx8p during meiosis should be different from its functional role in mitosis.

Slx8p can cooperate with Slx5p to work as a SUMO‐targeted E3 ubiquitin ligase responsible for the turnover of poly‐SUMOylated proteins.^25^ Since SUMOylated proteins accumulated in *slx8Δ* cells during meiosis, we speculated that Slx8p might regulate meiosis through its SUMO‐targeted ubiquitin ligase activity, which includes E3 ligase activity and the recognition of SUMO. To test this possibility, we first generated two vectors: *SLX8* and *SLX8* with a *RING* domain mutant (*SLX8^rm^*) under the control of its own promoter (Figure 4B). Each of the vectors was transformed into an *SLX8* deletion strain. The expression of *SLX8* in *slx8Δ* cells successfully rescued the sporulation defect (Figure 4C), whereas the expression of *SLX8^rm^* could not (Figure 4C). Moreover, we found that the spore viability of *SLX8^rm^* transformed cells was similar to that of the *slx8Δ* cells (Figure 4D), indicating that Slx8p participation in yeast meiosis is dependent on its E3 activity. Next, we determined whether Slx8p modulated meiosis through its SUMO‐interacting motif (SIM) (Figure 4B,E). As Slx5p also contributes to the SUMO binding activity of the Slx5p‐Slx8p complex,^25^ we transformed a SIM domain‐deleted mutant of *SLX8* into *slx5Δ slx8Δ* cells. We found that once the SUMO binding activity of Slx5p‐Slx8p was abolished, sporulation efficiency and spore viability could not be rescued (Figure 4E,F), which was a similar outcome with the RING domain mutant in Slx8p. Thus, both the E3 ligase activity and SUMO recognition of Slx8p are required for its functional roles during yeast meiosis, and Slx8p participation in meiosis is dependent on its SUMO‐targeted ubiquitin ligase activity.

### 2.7 Slx8p Facilitates SC Components Degradation via SUMO‐Ubiquitination Crosstalk

As many SC components could be modified by SUMO,^3^, ^7^, ^13^, ^37^, ^38^ including Red1p, Zip1p, and Ecm11p, and our results indicated Slx8p was required for SC degradation, we speculated that Slx8p might recognize the SUMOylated SC components and promote their ubiquitination to facilitate SC degradation. To test this possibility, we first examined the localization of Slx8p in chromatin spreading during meiotic prophase I. At the leptotene stage, Slx8p rarely loaded onto the chromosomal axis (Figure 4G). When homologous chromosomes were synapsed at the prophase stage, Slx8p was recruited to the SC and formed linear stretches or discontinuous signals that were co‐localized with Zip1p (Figure 4G). Next, we tried to discern whether this localization of Slx8p was dependent on its SIM that recognized SUMOylated proteins on the SC. As Slx5p and Slx8p may participate in meiosis by working as a heterodimeric complex, we transfected the *SLX8^simΔ^* into *slx5Δ slx8Δ* cells to exclude the influence of the SIM domain on Slx5p and endogenic Slx8p. We found the Slx8p SIM mutant was not recruited to the SC (Figure 4G). Thus, Slx8p might be recruited to the SC by recognizing the SUMOylated proteins via its SIM. The major function of Slx8p is to assemble polyubiquitin chains on SUMO or SUMOylated proteins, and Slx8p is also responsible for the turnover of SUMOylated proteins.^25^, ^39^ To evaluate this function, we directly fused a linear tetraubiquitin chain onto SUMO (Ub4‐Smt3) and transformed it in *slx8Δ* cells to test whether Slx8p mediated SUMO‐ubiquitination crosstalk is required for meiosis. We found the expression of this artificial Ub4‐SUMO hybrid partially rescued the sporulation defect in *slx8Δ* cells (Figure 4I–K), demonstrating that Slx8p mediated SUMO‐ubiquitination crosstalk was required for yeast meiosis. Therefore, Slx8p might recognize SUMOylated SC components and promote their ubiquitination to facilitate chromosome segregation.

### 2.8 Slx5p‐Slx8p Catalyzes the Ubiquitination of SUMOylated Zip1p and Ecm11p

**Figure 5:**
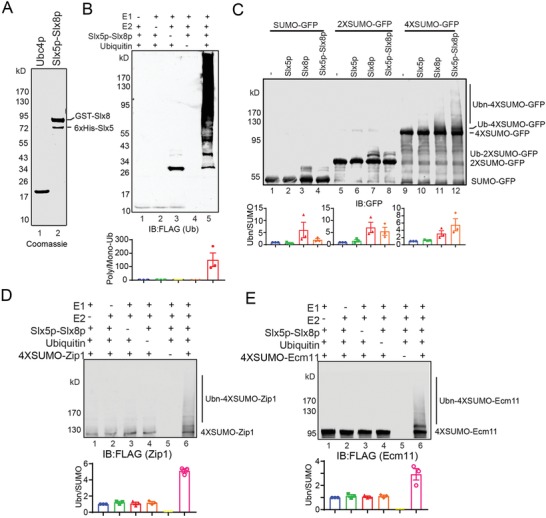
Slx5p‐Slx8p directly catalyze the ubiquitination on SUMOylated Zip1p and Ecm11p in vitro. A) Coomassie blue‐stained gels showing the expression and purification of Ubc4p and Slx5p‐Slx8p. B) Slx5p‐Slx8p has E3 ligase activity in vitro. The ubiquitination system was analyzed in the absence of E1, E2, Slx5p‐Slx8p, Ub, or all in vitro, which contained ubiquitination buffer and ATP. The relative amounts of Ub‐conjugates and mono‐Ub were quantified using Odyssey software (*n* = 3 independent experiments). Data are presented as mean ± SEM. C) Slx5p‐Slx8p directly catalyzed the polyubiquitination of the SUMOylated GFP. Either SUMO‐GFP, 2 × SUMO‐GFP, or 4 × SUMO‐GFP were added to the Slx5p, Slx8p, or Slx5p‐Slx8p ubiquitination system in vitro, which contained E1, E2, ubiquitin, ATP, Slx5p, Slx8p, or Slx5p‐Slx8p. The relative amounts of ubiquitinated SUMO‐GFP, 2 × SUMO‐GFP, 4 × SUMO‐GFP and free SUMO‐GFP, 2 × SUMO‐GFP, 4 × SUMO‐GFP were quantified using Odyssey software (*n* = 3 independent experiments). Data are presented as mean ± SEM. D,E) Slx5p‐Slx8p directly catalyzed the ubiquitination on SUMOylated Zip1p and Ecm11p in vitro. Either D) 4 × SUMO‐Zip1 or E) 4 × SUMO‐Ecm11 were added to the Slx5p‐Slx8p ubiquitination system in vitro, which contained E1, E2, Slx5p‐Slx8p, ubiquitin, and ATP. The relative amounts of ubiquitinated 4 × SUMO‐Zip1, 4 × SUMO‐Ecm11 and free 4 × SUMO‐Zip1, 4 × SUMO‐Ecm11 were quantified using Odyssey software (*n* = 3 independent experiments). Data are presented as mean ± SEM. See also Figure S7 in the Supporting Information.

We next wanted to identify the key substrate of Slx8p during meiosis. Among the three reported SUMOylated proteins of SC, we found the degradation of Zip1p and Ecm11p were affected in *slx8Δ* cells (Figure 3C–F); thus, we focused on Ecm11p and Zip1p for further investigation. First, we reconstituted an Slx5p‐Slx8p‐mediated in vitro ubiquitination system using purified components (**Figure** **5**A). When purified Slx5p, Slx8p, or Slx5p‐Slx8p was added to a reaction containing E1, Ubc4p, and ubiquitin, it efficiently enhanced polyubiquitination (Figure 5B; Figure S7A,B, Supporting Information), as previously demonstrated.^39^, ^40^ To further test whether Slx5p, Slx8p, or Slx5p‐Slx8p possess STUbL activity, we generated SUMO‐GFP, 2 × SUMO‐GFP, and 4 × SUMO‐GFP (Figure S7C, Supporting Information) and added them to the Slx5p, Slx8p, or Slx5p‐Slx8p ubiquitination system. We found that Slx5p, Slx8p, and Slx5p‐Slx8p could directly add ubiquitin to SUMO‐fused GFP. In detail, Slx5p only added ubiquitin to 4 × SUMO‐GFP; Slx8p preferred to catalyze monoubiquitination to SUMO‐fused GFP; Slx5p‐Slx8p could add multiubiquitin to 4 × SUMO‐GFP. Furthermore, the ubiquitination activity was dependent on the length of the SUMO chain on the substrates (Figure 5C). Next, we generated 4 × SUMO‐Zip1 and 4 × SUMO‐Ecm11, and added them to the Slx5p, Slx8p, or Slx5p‐Slx8p ubiquitination system. Slx5p could not add ubiquitin to either 4 × SUMO‐Zip1 or 4 × SUMO‐Ecm11. Similar to SUMO‐fused GFP, Slx8p could catalyze monoubiquitination, and Slx5p‐Slx8p could directly add multiubiquitin to 4 × SUMO‐Zip1 and 4 × SUMO‐Ecm11 (Figure 5D,E; Figure S7D–F, Supporting Information), indicating that Slx5p‐Slx8p directly catalyzes the ubiquitination of SUMOylated Zip1p and Ecm11p.

### 2.9 Ecm11p Is the Key Substrate of Slx8p during Meiosis

**Figure 6:**
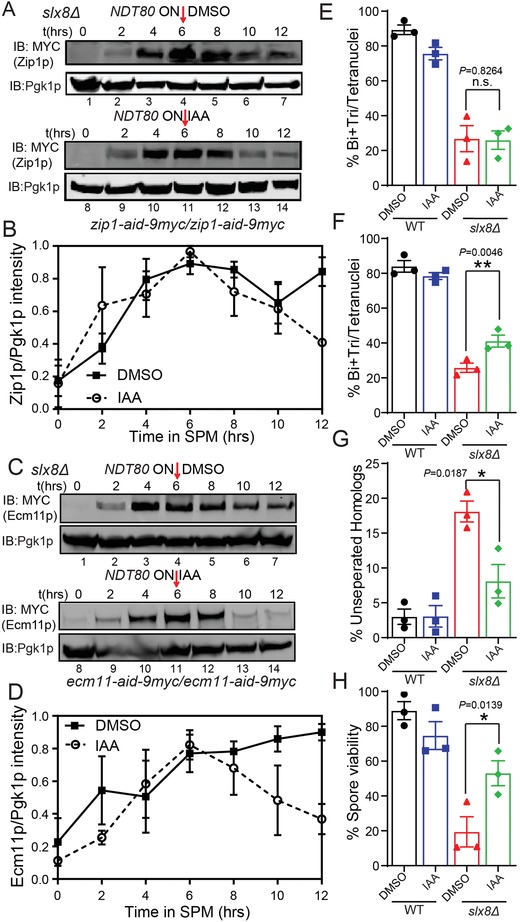
Ecm11p is the key substrate of Slx8p for accurate chromosome segregation. A–D) Conditional protein degradation of A) Zip1p and C) Ecm11p using the auxin‐induced degradation (AID) system in *slx8Δ* cells. Expression of A) Zip1p‐AID‐9 MYC and C) Ecm11p‐AID‐9 MYC were analyzed by immunoblotting using an anti‐MYC antibody. Pgk1p served as a loading control. Ratios of B) Zip1p/Pgk1p and D) Ecm11p/Pgk1p levels were normalized to the maximum Zip1/Pgk1p and Ecm11p/Pgk1p ratios (*n* = 3 independent experiments). Data are presented as mean ± SEM. E,F) The proportion of divided nuclei after induced Zip1p or Ecm11p degradation. E) WT *ZIP1‐AID‐9 × MYC*, *slx8Δ ZIP1‐AID‐9 × MYC* cells and F) WT *ECM11‐AID‐9 × MYC*, *slx8Δ ECM11‐AID‐9 × MYC* cells were analyzed for sporulation and released from the pachytene arrest by the addition of 1 × 10^−6^ m β‐estradiol at 6 h. Auxin in DMSO or DMSO alone was added at the same time. More than 150 nuclei were scored for each experiment (*n* = 3 independent experiments). Data are presented as mean ± SEM. **P < 0.01. Two‐sided *t*‐tests were used for statistical analyses. G) The proportion of mis‐separated chromosomes after induced Ecm11p degradation. More than 150 cells were scored for each experiment (*n* = 3 independent experiments). Data are presented as mean ± SEM. *P < 0.05. Two‐sided *t*‐tests were used for statistical analyses. H) The proportion of spore viability after induced Ecm11p degradation. More than 100 spores were scored for each experiment (*n* = 3 independent experiments). Data are presented as mean ± SEM. *P < 0.05. Two‐sided *t*‐tests were used for statistical analyses.

As Slx5p‐Slx8p is responsible for the turnover of SUMOylated proteins by promoting their ubiquitination,^25^, ^39^ and the degradation of Zip1p and Ecm11p was delayed in *slx8Δ* cells (Figure 3C–F), we speculated that Slx8p might promote the degradation of Zip1p and Ecm11p to promote meiotic progression. To test this possibility, we directly depleted Zip1p and Ecm11p proteins in *slx8Δ* cells via an auxin‐induced degradation (AID) system.^41^, ^42^ The AID system relies on an auxin‐induced interaction between the indole‐3‐acetic acid (IAA) family transcription factors and the F‐box protein TIR1, which results in the ubiquitination of AID‐fused proteins and their subsequent degradation by the proteasome.^42^ An AID‐tag was added (knocked‐in) to the C‐terminus of *ECM11* and *ZIP1* in both alleles of the WT and *slx8Δ* strains. The AID‐tag did not affect the meiotic progression of WT strains (**Figure** **6**E,F), suggesting that the tag did not influence the function of these proteins. Once IAA was added to the medium, both Ecm11p and Zip1p were degraded in *slx8Δ* cells (Figure 6A–D). After establishing this system, we next monitored the effect of induced degradation of Ecm11p and Zip1p on sporulation, finding that only the degradation of Ecm11p could partially rescue the sporulation defect of *slx8Δ* cells (Figure 6E,F). In addition, the chromosome missegregation defect and the spore viability of *slx8Δ* cells were all rescued to some extent (Figure 6G,H). These results suggest that Ecm11p is the key substrate of Slx8p, and Slx8p might add ubiquitin to SUMOylated Ecm11p to promote its degradation, thus facilitating meiotic progression.

## 3 Discussion

STUbLs have been reported to control the turnover of SUMOylated proteins via ubiquitin‐dependent protein degradation to ensure appropriate cellular levels of SUMOylated proteins.^43^ During mitosis, SUMOylated proteins are accumulated due to STUbLs deletion, which results in slight growth defects and decreased genotoxin resistance. These defects could be suppressed by the deletion of a SUMO ligase *SIZ1*.^36^ However, during meiosis, deletions of *SIZ1*, *SIZ2*, and *ZIP3* could not rescue the sporulation defect in *slx8Δ* cells (Figure S6, Supporting Information), indicating that Slx8p uses a different mechanism to regulate meiosis.

**Figure 7:**
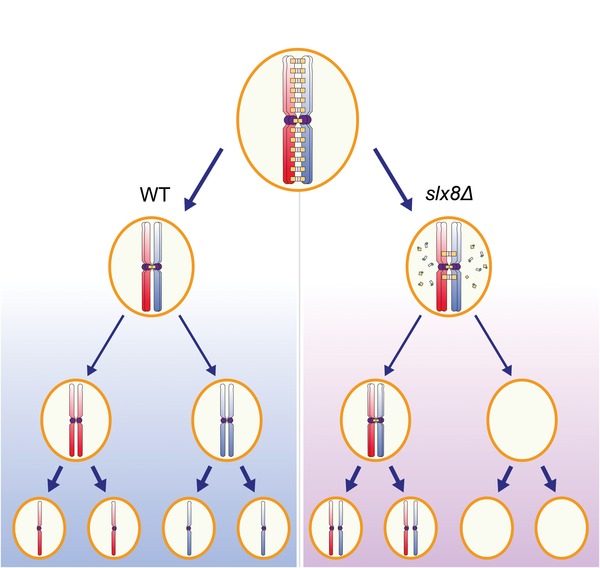
The functional role of Slx5p‐Slx8p in promoting accurate chromosome segregation by degrading SC components during meiosis. During meiosis, Slx5p‐Slx8p may coordinate ubiquitination and SUMOylation of SC components to promote their efficient degradation in both chromosome arms and centromeres. The efficient SC component degradation could facilitate proper chromosomal segregation. In *slx8Δ* cells, the degradation of SC components is perturbed during meiosis, and the persistent SC components in both chromosome arms and centromeres may impair accurate chromosome segregation and finally lead to aneuploidy.

The SC is a meiosis‐specific proteinaceous structure that stabilizes the interactions between homologous chromosomes during meiotic prophase I and allows for interhomolog crossover formation.^3^, ^6^, ^7^ Although the mechanism of SC assembly has been well established and is regulated by a combination of nonstructural proteins and PTMs, such as SUMOylation, phosphorylation, and N‐terminal acetylation,^3^, ^7^ the SC disassembly mechanism and the roles of PTMs remain poorly understood. The transition from prophase I to metaphase I, following SC disassembly, is a crucial event for equal distribution of genetic materials and genome stability. When cells exit from pachytene, the SCs could be disassembled on the chromosome arm region,^31^ while some may remain at centromeres to produce a functional tether for homologous chromosomes.^22^ It has been proposed that the degradation of SC components is essential for proper segregation of homologs during MI.^3^, ^7^ In the current study, we found that the disruption of *SLX8* mainly leads to chromosome missegregation (Figure 3). We also found that delayed SC component degradation is responsible for the meiotic defects in *slx8Δ* cells because forced degradation of the SC component partially rescued the chromosome segregation defect of the *slx8Δ* cells (Figure 6). It has been reported that both ubiquitin and proteasomes could directly load onto the chromosome axes and co‐localize with the SC during the late pachytene stage.^23^, ^24^ Consistent with these observation, we found that Slx8p‐Slx5p, a heterodimeric STUbL, recognized the SUMOylated SC components via their SIM domains for recruitment onto the SC; Slx8p‐Slx5p then directly added ubiquitin to SUMOylated Ecm11p and Zip1p to promote their degradation (Figures 4, 5, 6). The SUMO‐ubiquitination crosstalk is an economical way of displacing SC components from the corresponding chromosome regions, as it is unnecessary to resolve the SUMOylation before their degradation. Although SC disassembly in chromosome regions could not fully address the sporulation defect of the *SLX8* deletion, since disrupted SC formation in *spo11Δ slx8Δ* cells could not rescue its sporulation defect (Figure S4A, Supporting Information), the dotty Zip1p signal in *spo11Δ slx8Δ* cells was dramatically increased compared to that in *spo11Δ* cells (Figure S4D, Supporting Information). Moreover, the proportion of Zip1p PCs were significantly higher in *slx8Δ* cells than in the control groups (Figure S3, Supporting Information). This result suggests Slx5p‐Slx8p might also promote SC components degradation at the centromeres or even in some regions not associated with chromatin, thereby facilitating the homologous chromosome separation. Our findings demonstrate that persisting Zip1p is associated with chromosome missegregation, and Slx8p‐dependent degradation of SC component Ecm11p partially rescues the chromosome segregation defect. Slx5p‐Slx8p may coordinate ubiquitination and SUMOylation of SC components to promote their efficient degradation, thus facilitating proper chromosomal segregation (Figures 2, 3, and **7**; Figures S3–S5, Supporting Information).

Although far less is known about the mechanism of SC disassembly than SC assembly, some disassembly studies have provided enough details to advance our understanding of this process. In yeast, Cdc5p and Ipl1p have been shown to be essential for pachytene exit and SC destruction.^36^, ^44^ Ipl1p could coordinate synaptonemal complex disassembly with resolution of crossovers and cell cycle progression at the end of meiotic prophase. Cdc5p, the only Polo‐like kinase in budding yeast, is regulated by Ndt80p and controls pachytene exit.^45^ The expression of Cdc5p triggers an untimely disassembly of the SC and irregular resolution of recombination intermediates.^30^ Cdc5p could collaborate with CDK1 to hyperphosphorylate Dbf4p, the regulatory subunit of Dbf4p‐dependent Cdc7p kinase (DDK), and hyperphosphorylated DDK greatly accelerates SC destruction.^46^ However, the substrates of Cdc5p, Dbf4p, and Ipl1p, as well as the precise mechanism underlying their triggering of SC disassembly, remain unknown. We noticed that the protein level of Ndt80p was increased in *slx8Δ* cells (Figure 2E), suggesting that Cdc5p should be continuously induced by Ndt80p and promote SC disassembly in *SLX8* deleted cells. Contrary to this expectation, degradation of SC components was delayed rather than accelerated in *slx8Δ* cells (Figure 3C–F). Thus, Slx8p‐dependent SC degradation appears to occur downstream of the Cdc5p‐promoted process. Further studies are needed to explore the detailed relationships between Slx5p‐Slx8p and these reported SC disassembly factors.

The artificial Ub4‐SUMO hybrid and the induced degradation of Ecm11p could only partially rescue the sporulation defect in *slx8Δ* cells, indicating Slx8p may play multiple roles during meiosis. We found that premeiotic DNA replication showed a minor delay and the expression of some SC components and meiotic cohesin was impaired to some extent in *slx8Δ* cells (Figures 1G,J,K and 3A–F). In addition, Slx5p‐Slx8p has been reported to modulate the crossover interference during meiosis.^47^ Therefore, future efforts are still needed to address the functional roles of Slx5p‐Slx8p in other aspects of meiosis.

## 4 Experimental Section

*Antibodies and Proteins*: The FLAG (1:2000, M20008L), MYC (1:1000, M20002M), and mouse GFP (1:1000, M20004M) antibodies were purchased from Abmart (Shanghai, China). The rabbit FLAG (1:200, PM020) antibody was purchased from MBL (Nagoya, Japan). The goat Zip1 (1:100, sc‐15632) antibody was purchased from Santa Cruz Biotechnologies (Dallas, USA). The Pgk1 (1:5000) and GFP (1:200) polyclonal antibodies were generated in rabbits using the corresponding recombinant proteins as antigens. The E1 enzyme and ubiquitin were purchased from Boston Biochem (Cambridge, MA).

*Strains and Plasmids*: All yeast strains and plasmids used in this study are described in Tables S1 and S2 in the Supporting Information.

*Sporulation Conditions and Meiotic Nuclear Division Assays*: Sporulation was induced using potassium acetate as previously described.^48^ The strains were grown for 24 h in YPD medium (1% yeast extract, 2% peptone, and 2% glucose), diluted in liquid YPA medium (1% yeast extract, 2% peptone, and 2% potassium acetate) to OD600 = 0.3, and grown for 10 h at 30 °C. Cells were harvested, washed, and resuspended in a sporulation medium (SPM, 2% potassium acetate) to OD600 = 1.9, and sporulated at 30 °C for different durations. Meiotic nuclear divisions were visualized by staining chromosomal DNA with 1 µg mL^−1^ 4′,6′‐diamidino‐2‐phenylindole (DAPI): the samples were harvested at the indicated times and directly fixed in an equal volume of 100% ethanol for subsequent DAPI staining. Images were recorded and analyzed using a Nikon Eclipse Ti microscope (Nikon, Eclipse Ti‐S, Tokyo, Japan). Spore viability was determined by dissection of tetrads from SPM that had been incubated for 3–4 days at 30 °C.

*Immunoblotting*: Cells were treated with mild alkaline regents and boiled in a standard electrophoresis loading buffer, as previously described.^49^ The samples were separated by sodium dodecyl sulfate polyacrylamide gel electrophoresis (SDS‐PAGE) under reducing conditions and transferred to nitrocellulose membranes. The blots were incubated with a primary antibody and a fluorescent dye‐labeled secondary antibody (926‐32211, LI‐COR, Lincoln, NE); the blots were then scanned using an Odyssey infrared 740 imager (9120, LI‐COR Biosciences, Lincoln, NE).

*Flow Cytometry*: To analyze the DNA content, 1 × 10^7^ cells were fixed with 1 mL cold 70% ethanol overnight and then resuspended in 1 mL of 50 × 10^−3^ m sodium citrate. The samples were centrifuged at 2000 rpm for 5 min, and the supernatant was removed. The pellets were digested with RNase A for 2 h at 37 °C and then sonicated for 2 s at 20% power, stained with 1 × 10^−6^ m Sytox Green (Molecular Probes, S‐7020), and analyzed using a BD FACSvantage SE Flow Cytometry System (Franklin Lakes, NJ).

*Meiotic Surface Spread Nuclei*: Meiotic chromosome spread, staining, and imaging were carried out as previously described^47^ with the following modifications: 80 µL 1× MES and 200 µL 4% paraformaldehyde were added to spheroplasted, washed cells, and then cells were lysed and spread on a glass microscope slide with 1% Lipsol (LIP Ltd., Shipley England) and fixed by 3% w/v paraformaldehyde with 3.4% w/v sucrose as described.^47^ The slide was allowed to air dry until less than half of the liquid remained, and then washed in 0.4% Photo‐flo as described.^50^ Primary antibodies were added to the sections and incubated at 4 °C overnight, followed by incubation with the secondary antibodies. The nuclei were stained with DAPI. The images were taken immediately using an LSM 780 microscope (Zeiss, Oberkochen, Germany) or a TCS SP8 microscope (Leica, Wetzlar, Germany). To identify meiotic nuclei at prophase I, dotty, dot‐linear, and full linear Zip1‐GFP staining were used. For subsequent stages, mCherry‐tubulin and *CNM67*‐GFP were used to assess spindle and SPB behavior.

*Homologous Chromosome Segregation Assay*: A homologous chromosome segregation assay was performed as previously described.^34^ The tetO array was inserted into the *HIS4* locus consisting of 120 DNA fragments; each fragment contained the 19‐bp tetO binding site and a unique 10‐bp spacer. The *pTetR‐kGFP* plasmid was constructed by PCR using the *pTetR‐GFP* plasmid, which expresses TetR‐GFP fusion protein under the control of the *URA3* promoter. The plasmid was cut with AflII and integrated into the *LEU2* locus. Microscopy was performed on an inverted microscope (Nikon, Tokyo, Japan) with a 100× oil immersion objective.

*Protein Purification*: Slx5p, Slx8p, Slx5p‐Slx8p, Ubc4p, SUMO‐GFP, 2 × SUMO‐GFP, 4 × SUMO‐GFP, 4 × SUMO‐Zip1p, and 4 × SUMO‐Ecm11p were expressed as N‐terminal hexahistidine‐tagged or Glutathione S‐transferase (GST)‐tagged fusion proteins in pET28a or pGEX‐4t‐1 vectors. Briefly, the plasmid was introduced into Rosetta (DE3) cells and grown in Terrific Broth at 37 °C to an OD600 of 0.8. The temperature of the culture was then shifted to 16 °C, and cells were induced with 0.25 × 10^−3^ m isopropyl‐d‐thiogalactoside (IPTG) for 16 h. Cells were harvested by centrifugation and resuspended in lysis buffer (20 × 10^−3^ m Tris, pH 7.4, 300 × 10^−3^ m NaCl, 10 × 10^−3^ m imidazole, and 10% glycerol for hexahistidine‐tagged fusion protein; 50 × 10^−3^ m Tris, pH 7.4, 300 × 10^−3^ m NaCl, 2 × 10^−3^ m MgCl_2_, and 5% glycerol for GST‐tagged fusion protein) with 1 × 10^−3^ m PMSF. After lysis by sonication and high‐speed centrifugation of lysates, the supernatant was incubated with Ni Sepharose 6 Fast Flow (GE Healthcare, Marlborough, MA) or Glutathione Sepharose 4B (GE Healthcare, Marlborough, MA) for 2 h at 4 °C. The resin was washed, and the protein was eluted using the lysis buffer supplemented with 250 × 10^−3^ m imidazole or 10 × 10^−3^ m glutathione. Purified proteins were refolded in PBS and dialyzed in the aforementioned protein purification buffer.

*In Vitro Ubiquitination Assay*: E1 (60 × 10^−9^ m), Ubc4p (200 × 10^−9^ m), Slx5p (300 × 10^−9^ m), Slx8p (300 × 10^−9^ m) or Slx5p‐Slx8p (300 × 10^−9^ m), and SUMO‐GFP (400 × 10^−9^ m) were incubated with Ub (10 × 10^−9^ m) at 30 °C in a buffer containing 25 × 10^−3^ m Tris‐HCl (pH 7.4), 2 × 10^−3^ m magnesium/ATP, and 0.1 × 10^−3^ m DTT. Ubiquitination of SUMO‐GFP was detected by immunoblotting with an anti‐GFP antibody. Ubiquitination of 4 × SUMO‐Zip1p and 4 × SUMO‐Ecm11p was detected by immunoblotting with an anti‐MYC antibody. The immunoblotting was performed using a fluorescent dye‐labeled secondary antibody (Invitrogen, Carlsbad, CA), and the blots were scanned using an Odyssey infrared imager.

*Conditional Protein Degradation System Induced by Auxin*: The conditional protein degradation system induced by auxin was established as previously described.^41^ The C‐terminus of Zip1p and Ecm11p was fused to an AID tag together with 9MYC in all alleles. The gene encoding the AID‐specific E3 ubiquitin ligase OsTIR1 under the control of the *pADH1* promoter was constitutively expressed from a centromeric vector (pRS314 with *KANMX4*). Auxin (3‐indolo acetic acid, Sigma I5148) was dissolved in DMSO at 285 × 10^−3^ m. Synchronized sporulation strains were gifts from Angelika Amon,^51^ and the sporulation was performed as previously described.^51^ *GAL‐NDT80 GAL4.ER* strains were released from pachytene arrest by the addition of 1 × 10^−6^ m β‐estradiol (5 × 10^−3^ m stock in ethanol; Sigma, E2758‐1G) at 6 h and auxin was added to a final concentration of 0.5 × 10^−3^ m.

*Statistical Analysis*: All data were performed with at least three biological replicates, and a representative result is shown in the figures. All data were presented as the mean ± SEM. The statistical significance of the differences between the mean values for the different groups was measured by the Student's *t*‐test with a paired two‐tailed distribution. Categorical data were compared by the chi‐square test. *G*‐test was used for homogeneity. The data were considered significant when the *P* value was less than 0.05 (*) or 0.01 (**).

## Conflict of Interest

The authors declare no conflict of interest.

## Author Contributions

C.L., H.Z., and S.X. performed most of the experiments. T.H. and Y.C. performed the sporulation and tetrad analysis. T.W. and Y.M. performed the meiotic surface spreading experiment. S.X. and H.G. performed and analyzed some biochemical experiments. Z.X., L.L.D., J.L., G.L., and W.L. designed the experiments. G.L. and W.L. supervised the project and wrote the manuscript.

## Supporting information

Supporting Information

Supplemental Table 1
