# Supplementary material for: Slx5p‐Slx8p Promotes Accurate Chromosome Segregation by Mediating the Degradation of Synaptonemal Complex Components during Meiosis
Source: Adv Sci (Weinh). 2020 Jan 1;7(4):1900739. doi: 10.1002/advs.201900739 (PMC7029635; doi:10.1002/advs.201900739)
Supplement: Supplementary file 1 — Supporting Information [file ADVS-7-1900739-s001.pdf]

Copyright WILEY-VCH Verlag GmbH & Co. KGaA, 69469 Weinheim, Germany, 2019.

ADVANCED SCIENCE
Open Access

Supporting Information

for *Adv. Sci.,* DOI: 10.1002/advs.201900739

Slx5p-Slx8p Promotes Accurate Chromosome Segregation by Mediating the Degradation of Synaptonemal Complex Components during Meiosis

Chao Liu, Haichao Zhao, Sai Xiao, Tingting Han, Yinghong Chen, Tong Wang, Yanjie Ma, Hui Gao, Zhiping Xie, Li-Lin Du, Jian Li, Guoping Li, and Wei Li**

Supporting Information

Slx5p-Slx8p promotes accurate chromosome segregation by mediating the degradation of synaptonemal complex components during meiosis

Chao Liu, Haichao Zhao, Sai Xiao, Tingting Han, Yinghong Chen, Tong Wang, Yanjie Ma, Hui Gao, Zhiping Xie, Li-Lin Du, Jian Li, Guoping Li, Wei Li**

E-mail: **liguoping4195@bjhmoh.cn; leways@ioz.ac.cn**

Figures

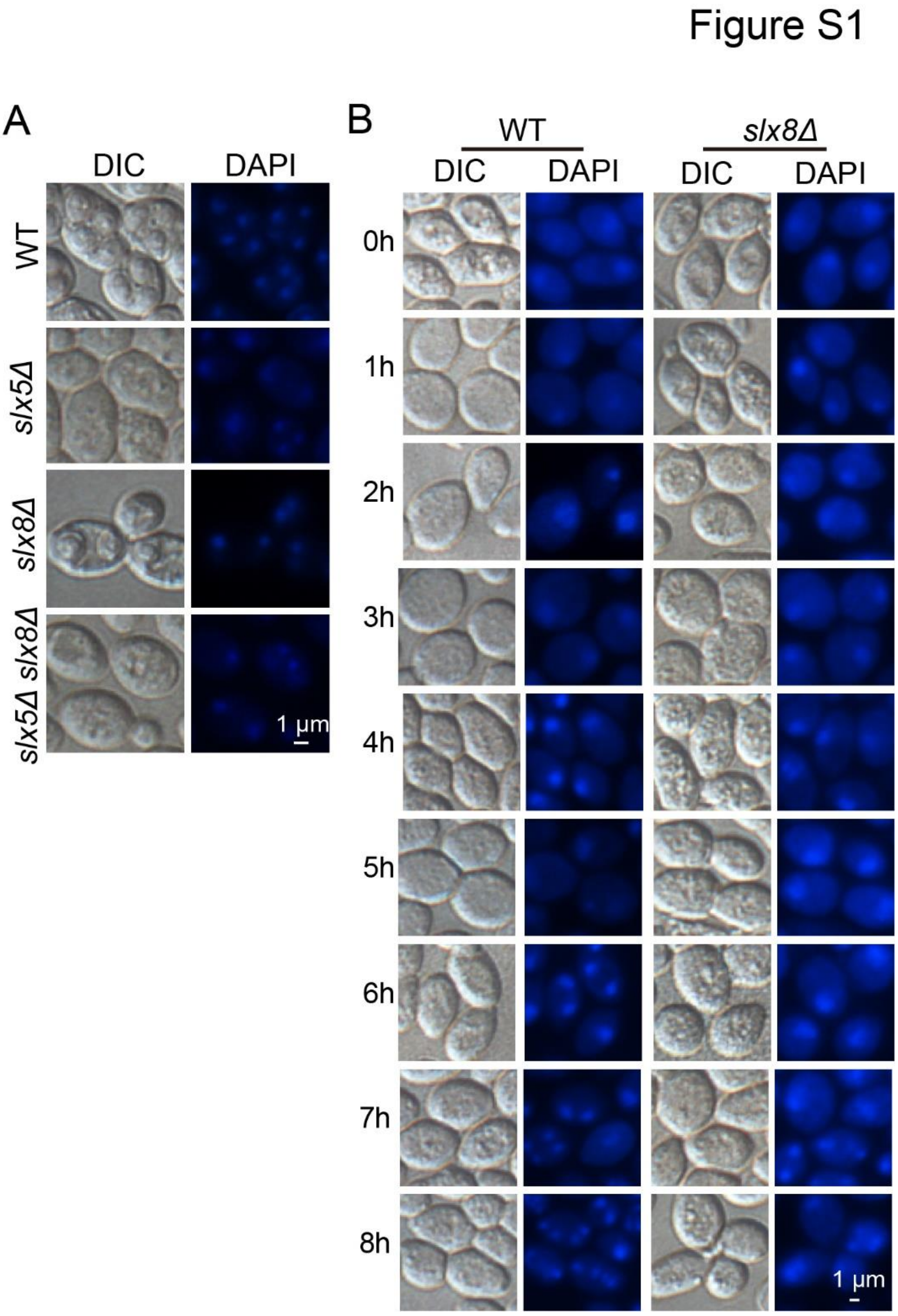

Figure S1 The disruption of *SLX5* and *SLX8* affect yeast sporulation

(**A**) WT, *slx8Δ, slx8Δ* and *slx5Δ slx8Δ* spores were stained with DAPI to show the decrease of sporulation efficiency.

(**B**) WT and *slx8Δ* spores were stained with DAPI at different time point during sporulation.

Figure S2

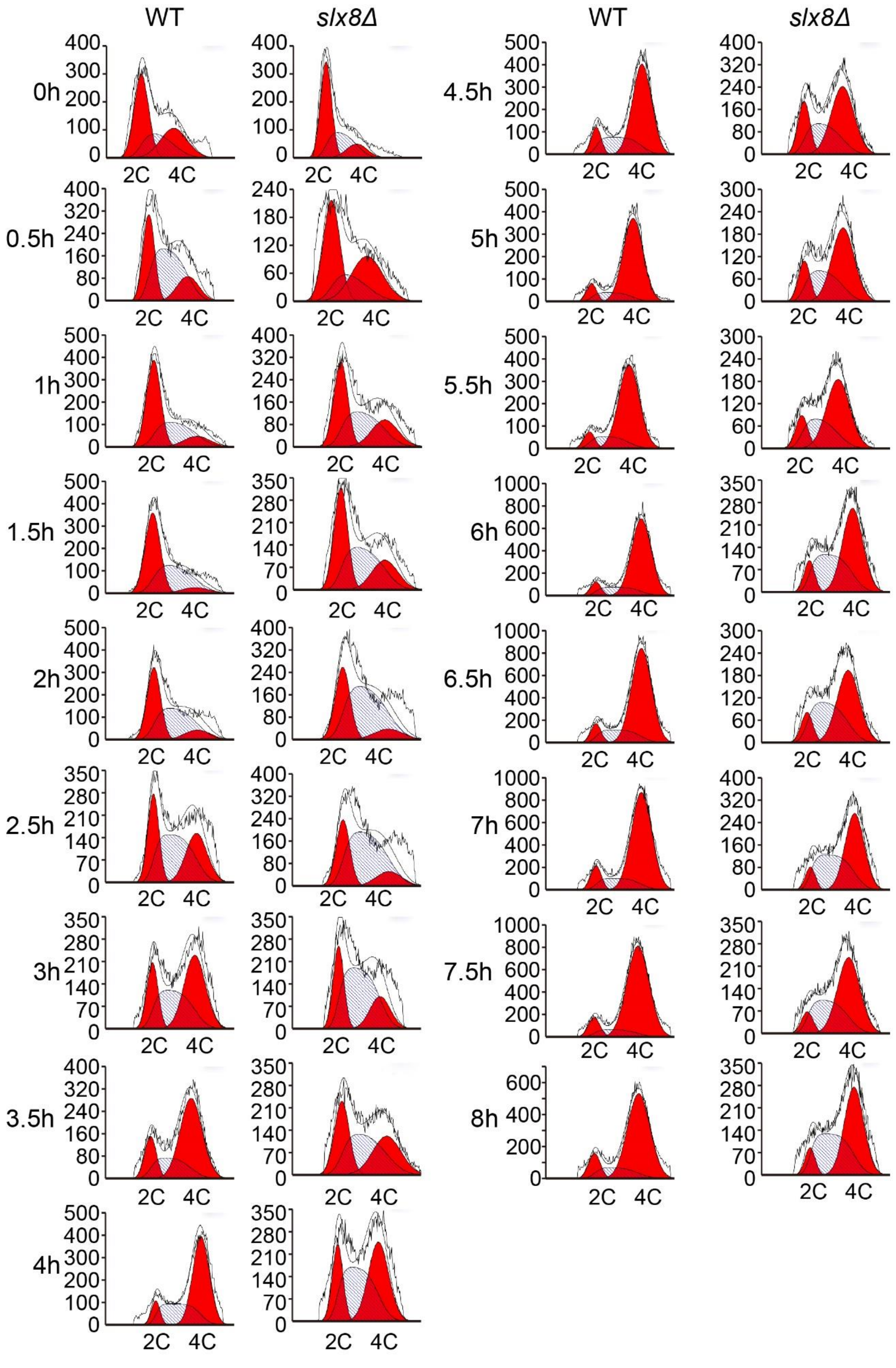

Figure S2 Pre-meiotic DNA replication was a little delay in the absence of *SLX8* during sporulation.

Figure S3

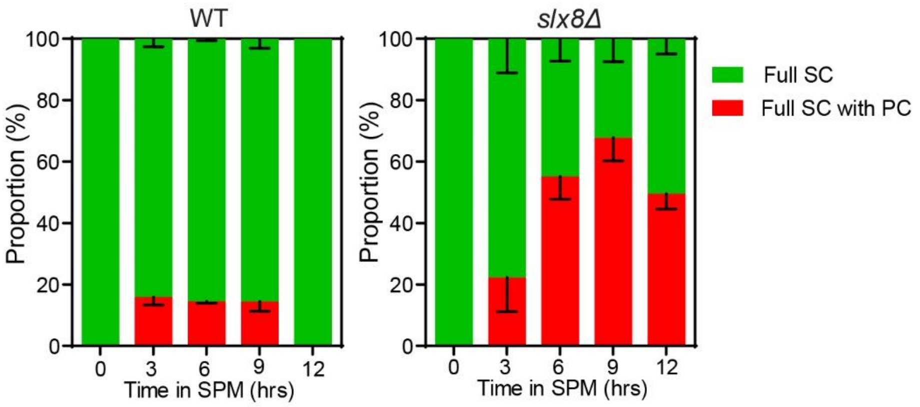

Figure S3 Proportion of nuclei with Zip1p aggregates (PC) in WT and *slx8Δ* cells.

More than 200 nuclei were scored for each time point (n=3 independent experiments). Data are presented as mean ± SEM.

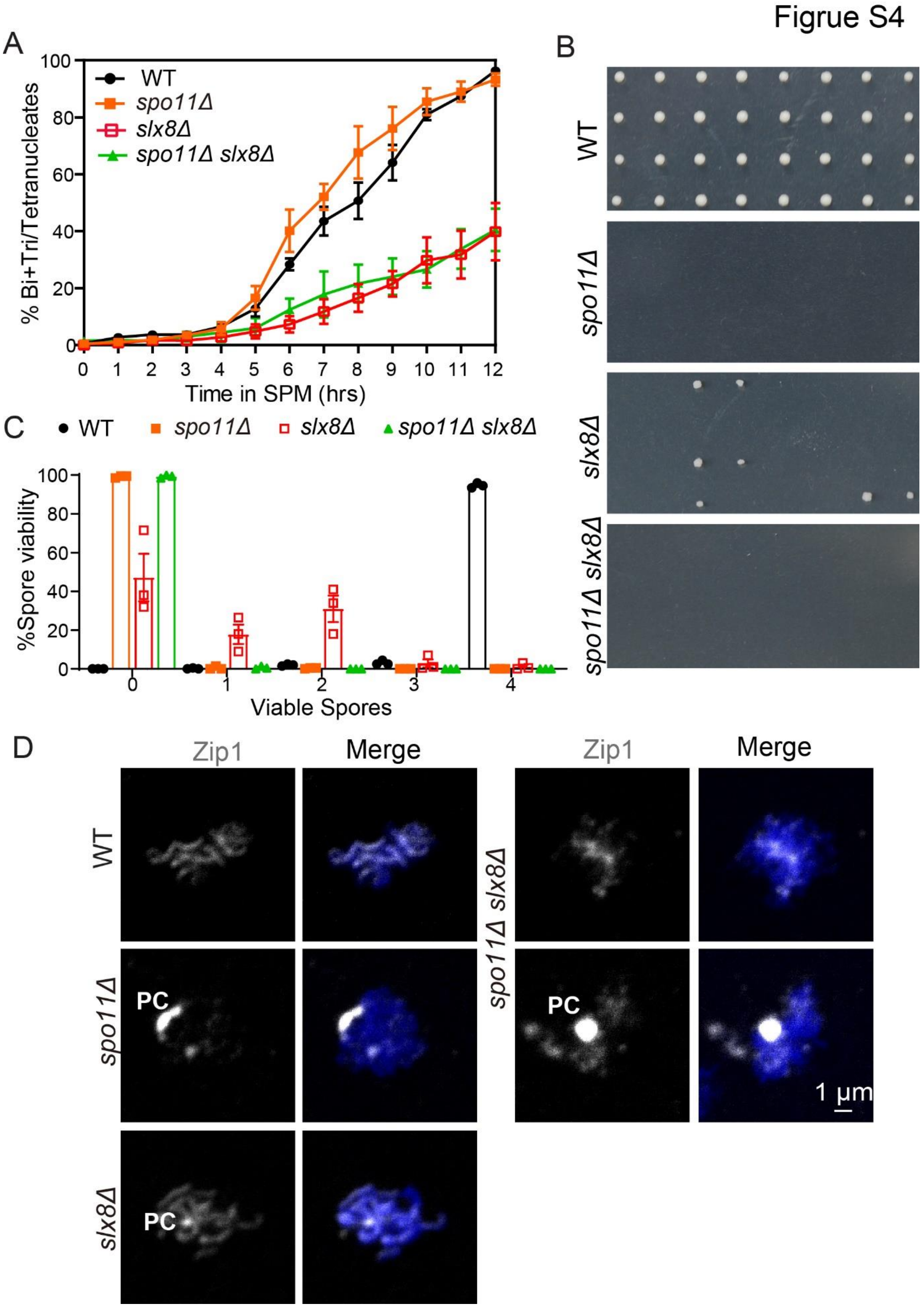

Figure S4 Meiosis delay conferred by the *SLX8* depletion cannot be rescued by *SPO11* deletion.

(**A**) Meiotic progression in WT, *slx8Δ*, *spo11Δ* and *spo11Δ slx8Δ* cells. More than 300 nuclei were scored for each experiment (n=3 independent experiments). Data are presented as mean ± SEM. Two-sided *t*-tests were used for statistical analyses.

(**B**) The spore viability in WT, *slx8Δ*, *spo11Δ* and *spo11Δ slx8Δ* cells. Tetrad analysis of WT, *slx8Δ*, *spo11Δ* and *spo11Δ slx8Δ* diploids were performed by dissection of tetrads after sporulation.

(**C**) The proportion of spore viability in WT, *slx8Δ*, *spo11Δ* and *spo11Δ slx8Δ* cells. More than 200 tetrads were scored for each experiment (n=3 independent experiments). Data are presented as mean ± SEM.

(**D**) Dotty Zip1p signal was dramatically increased in *spo11Δ slx8Δ* cells compared with that in *spo11Δ* cells

Figure S5

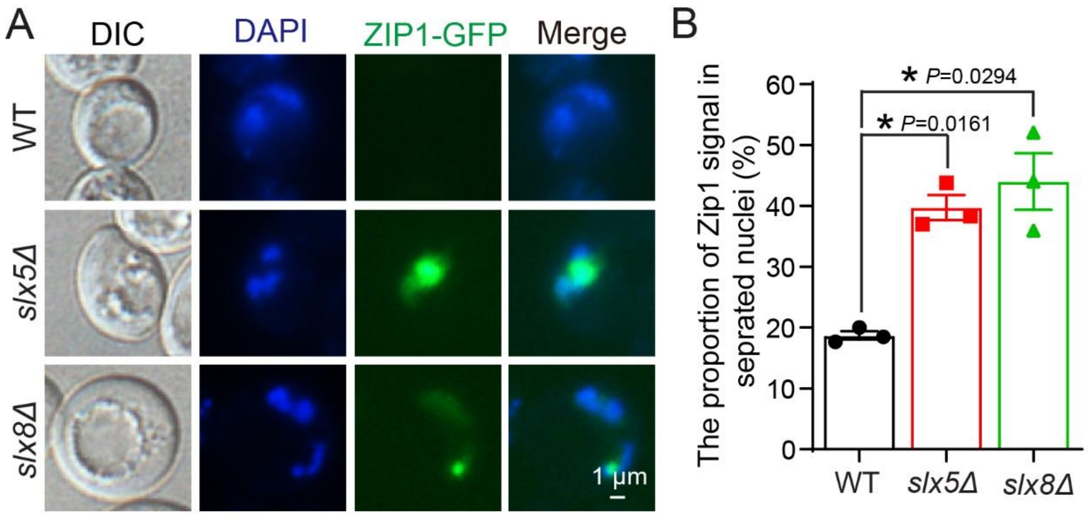

Figrue S5 Diffused Zip1p signal was retained in the separated nuclei in *slx5Δ*, *slx8Δ* cells

(**A**) WT, *slx5Δ* and *slx8Δ* carrying *pZip1-GFP*^{700} were stained with DAPI.

(**B**) The proportion of Zip1 signal in separated nuclei. More than 120 cells were scored for each experiment (n=3 independent experiments). Data are presented as mean ± SEM. **P* < 0.05. Two-sided *t*-tests were used for statistical analyses.

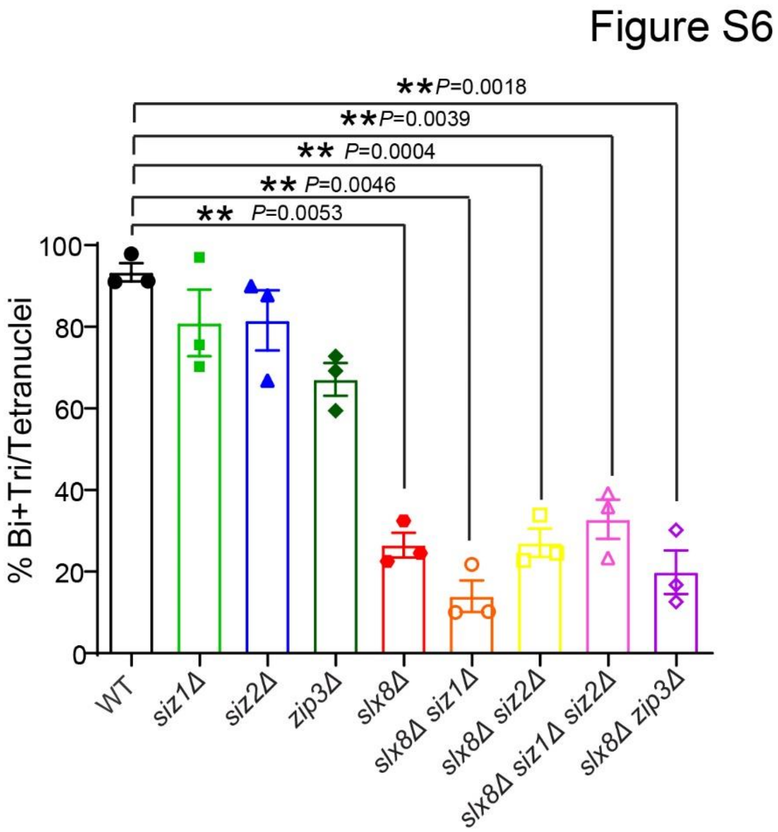

Figure S6 The deletion of SUMO ligase could not rescue the sporulation defect of *slx8Δ* cells.

The proportion of divided nucleates in WT, *siz1Δ*, *siz2Δ*, *zip3Δ*, *slx8Δ*, *slx8Δ siz1Δ*, *slx8Δ siz2Δ*, *slx8Δ siz1Δ siz2Δ*, and *slx8Δ zip3Δ*cells. More than 170 nuclei were scored for each experiment (n=3 independent experiments). Data are presented as mean ± SEM. ***P* < 0.01. Two-sided *t*-tests were used for statistical analyses.

Figure S7

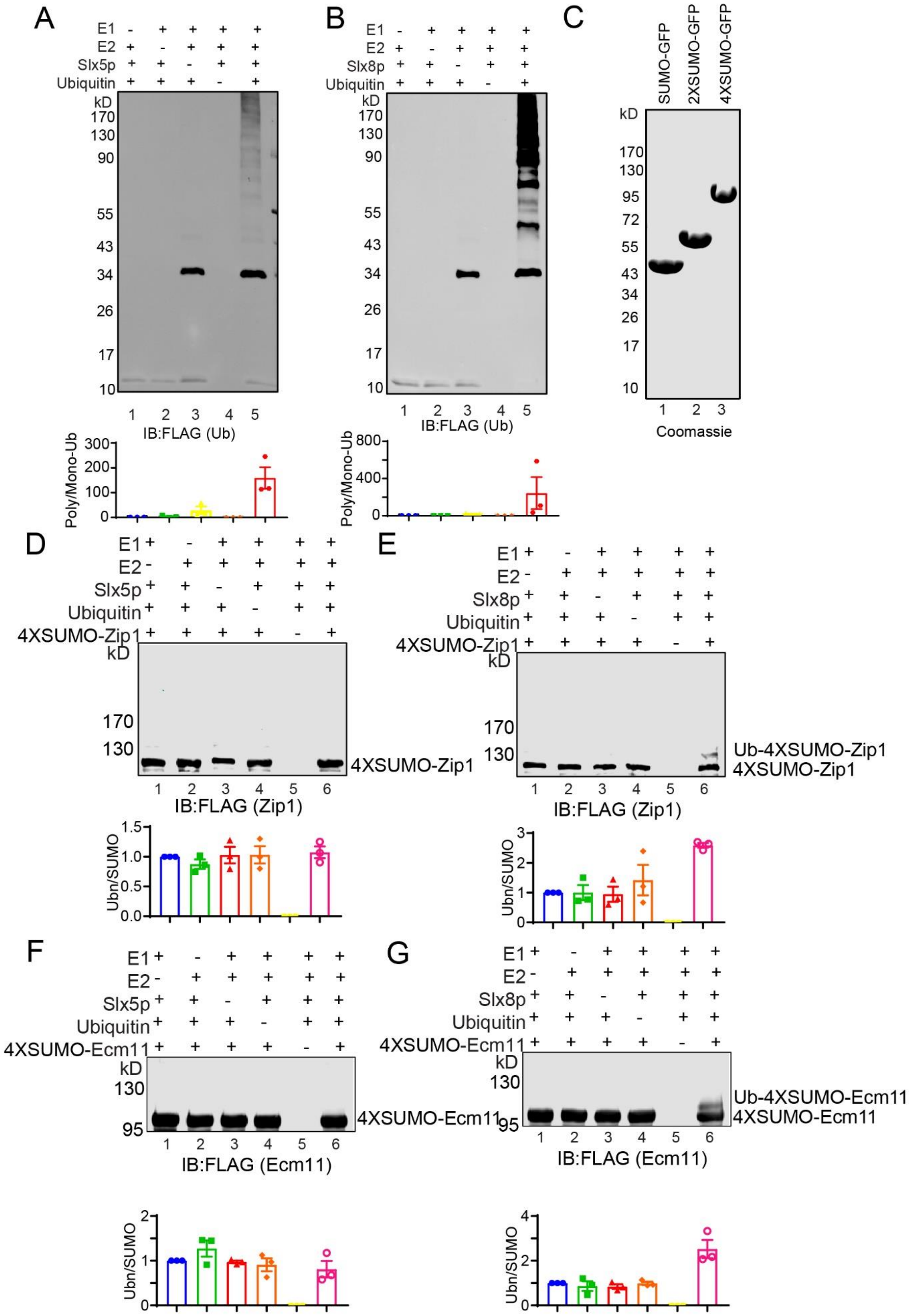

Figure S7 Reconstitute the Slx5p, Slx8p-mediated ubiquitination *in vitro*.

(**A**) and (**B**) Slx5p, Slx8p has E3 ligase activity *in vitro*. Either without E1, E2, Slx5p or Slx8p, Ub or all were added to the ubiquitination system *in vitro*, which contained ubiquitination buffer and ATP.

(**C**) Coomassie blue-stained gels showing the expression and purification of SUMO-GFP, 2×SUMO-GFP and 4×SUMO-GFP.

(**D**) and (**F**) Slx5p could not directly catalyse the ubiquitination on SUMOylated Zip1p and Ecm11p *in vitro*. Either 4×SUMO-Zip1 (**D**) or 4×SUMO-Ecm11 (**F**) were added to the Slx5p ubiquitination system *in vitro*, which contained E1, E2, Slx5p, ubiquitin and ATP.

(**E**) and (**G**) Slx8p could directly catalyse the ubiquitination on SUMOylated Zip1p and Ecm11p *in vitro*. Either 4×SUMO-Zip1 (**E**) or 4×SUMO-Ecm11 (**G**) were added to the Slx8p ubiquitination system *in vitro*, which contained E1, E2, Slx8p, ubiquitin and ATP.

Tables

Table S1. Strains Used in This Study

Strain	Genotype	Source
LW0066	*MATa/α, ho::LYS2/ ho::LYS2, lys2/ lys2, ura3/ ura3, leu2::hisG/leu2::hisG, his3::hisG/ his3::hisG, trp1::hisG/ trp1::hisG*	[1]
LW1103	*MATa/α, ho::LYS2/ ho::LYS2, lys2/ lys2, ura3/ ura3, leu2::hisG/leu2::hisG, his3::hisG/ his3::hisG, trp1::hisG/ trp1::hisG* ***slx8::klTRP1/slx8::klTRP1***	This study
LW1106	*MATa/α, ho::LYS2/ ho::LYS2, lys2/ lys2, ura3/ ura3, leu2::hisG/leu2::hisG, his3::hisG/ his3::hisG, trp1::hisG/ trp1::hisG* ***uls1::KANMX4/uls1::KANMX4***	This study
LW1112	*MATa/α, ho::LYS2/ ho::LYS2, lys2/ lys2, ura3/ ura3, leu2::hisG/leu2::hisG, his3::hisG/ his3::hisG, trp1::hisG/ trp1::hisG* ***slx5::LEU2/slx5::LEU2***	This study
LW1115	*MATa/α, ho::LYS2/ ho::LYS2, lys2/ lys2, ura3/ ura3, leu2::hisG/leu2::hisG, his3::hisG/ his3::hisG, trp1::hisG/ trp1::hisG* ***slx5::KANMX4; slx8::klTRP1/ slx5::KANMX4; slx8::klTRP1***	This study
LW1121	*MATa/α, ho::LYS2/ ho::LYS2, lys2/ lys2, ura3/ ura3, leu2::hisG/leu2::hisG, his3::hisG/ his3::hisG, trp1::hisG/ trp1::hisG* ***rad18::KANMX4/rad18::KANMX4***	This study
LW1127	*MATa/α, ho::LYS2/ ho::LYS2, lys2/ lys2, ura3/ ura3, leu2::hisG/leu2::hisG, his3::hisG/ his3::hisG, trp1::hisG/ trp1::hisG* ***zip3::KANMX4/zip3::KANMX4***	This study

LW1130	*MATa/α, ho::LYS2/ ho::LYS2, lys2/ lys2, ura3/ ura3, leu2::hisG/leu2::hisG, his3::hisG/ his3::hisG, trp1::hisG/ trp1::hisG*<br>***zip3::KANMX4; slx8::klTRP1/zip3::KANMX4; slx8::klTRP1***	This study
LW1133	*MATa/α, ho::LYS2/ ho::LYS2, lys2/ lys2, ura3/ ura3, leu2::hisG/leu2::hisG, his3::hisG/ his3::hisG, trp1::hisG/ trp1::hisG*<br>***siz1::KANMX4/siz1::KANMX4***	This study
LW1136	*MATa/α, ho::LYS2/ ho::LYS2, lys2/ lys2, ura3/ ura3, leu2::hisG/leu2::hisG, his3::hisG/ his3::hisG, trp1::hisG/ trp1::hisG*<br>***siz1::KANMX4; slx8::klTRP1/siz1::KANMX4; slx8::klTRP****1*	This study
LW1139	*MATa/α, ho::LYS2/ ho::LYS2, lys2/ lys2, ura3/ ura3, leu2::hisG/leu2::hisG, his3::hisG/ his3::hisG, trp1::hisG/ trp1::hisG*<br>***siz2::KANMX4/siz2::KANMX4***	This study
LW1142	*MATa/α, ho::LYS2/ ho::LYS2, lys2/ lys2, ura3/ ura3, leu2::hisG/leu2::hisG, his3::hisG/ his3::hisG, trp1::hisG/ trp1::hisG*<br>***siz2::KANMX4; slx8::klTRP1/siz2::KANMX4; slx8::klTRP1***	This study
LW1145	*MATa/α, ho::LYS2/ ho::LYS2, lys2/ lys2, ura3/ ura3, leu2::hisG/leu2::hisG, his3::hisG/ his3::hisG, trp1::hisG/ trp1::hisG*<br>***siz1::KANMX4; siz2::LEU2; slx8::klTRP1/siz1::KANMX4; siz2::LEU2; slx8::klTRP1***	This study
LW1147	*MATa/α, ho::LYS2/ ho::LYS2, lys2/ lys2, ura3/ ura3, leu2::hisG/leu2::hisG, his3::hisG/ his3::hisG, trp1::hisG/ trp1::hisG*<br>***SLX8-9MYC****-HIS3/SLX8*	This study
LW1150	*MATa/α, ho::LYS2/ ho::LYS2, lys2/ lys2, ura3/ ura3, leu2::hisG/leu2::hisG, his3::hisG/ his3::hisG, trp1::hisG/ trp1::hisG*	This study

	spo11::KANMX4; slx8::klTRP1 / spo11::KANMX4; slx8::klTRP1	
LW1156	*MATa/α, ho::LYS2/ ho::LYS2, lys2/ lys2, ura3/ ura3, leu2::hisG/leu2::hisG, his3::hisG/ his3::hisG, trp1::hisG/ trp1::hisG*<br><br>***MCD1-9MYC****-HIS3/MCD1*	This study
LW1158	*MATa/α, ho::LYS2/ ho::LYS2, lys2/ lys2, ura3/ ura3, leu2::hisG/leu2::hisG, his3::hisG/ his3::hisG, trp1::hisG/ trp1::hisG*<br><br>***MCD1-9MYC****-HIS3/MCD1****; slx8::klTRP1 /slx8::klTRP1***	This study
LW1160	*MATa/α, ho::LYS2/ ho::LYS2, lys2/ lys2, ura3/ ura3, leu2::hisG/leu2::hisG, his3::hisG/ his3::hisG, trp1::hisG/ trp1::hisG*<br><br>***REC8-9MYC****-HIS3/REC8*	This study
LW1162	*MATa/α, ho::LYS2/ ho::LYS2, lys2/ lys2, ura3/ ura3, leu2::hisG/leu2::hisG, his3::hisG/ his3::hisG, trp1::hisG/ trp1::hisG*<br><br>***REC8-9MYC****-HIS3/REC8****; slx8::klTRP1 /slx8::klTRP1***	This study
LW1168	*MATa/α, ho::LYS2/ ho::LYS2, lys2/ lys2, ura3/ ura3, leu2::hisG/leu2::hisG, his3::hisG/ his3::hisG, trp1::hisG/ trp1::hisG*<br><br>***RED1-9MYC****-HIS3/RED1*	This study
LW1170	*MATa/α, ho::LYS2/ ho::LYS2, lys2/ lys2, ura3/ ura3, leu2::hisG/leu2::hisG, his3::hisG/ his3::hisG, trp1::hisG/ trp1::hisG*<br><br>***RED1-9MYC****-HIS3/RED1****; slx8::klTRP1 /slx8::klTRP1***	This study
LW1172	*MATa/α, ho::LYS2/ ho::LYS2, lys2/ lys2, ura3/ ura3, leu2::hisG/leu2::hisG, his3::hisG/ his3::hisG, trp1::hisG/ trp1::hisG*<br><br>***ZIP1-9MYC****-HIS3/ZIP1*	This study
LW1174	*MATa/α, ho::LYS2/ ho::LYS2, lys2/ lys2, ura3/ ura3, leu2::hisG/leu2::hisG, his3::hisG/ his3::hisG, trp1::hisG/*	This study

	trp1::hisG<br>***ZIP1-9MYC**-HIS3/ZIP1**; slx8::klTRP1 /slx8::klTRP1***	
LW1176	*MATa/α, ho::LYS2/ ho::LYS2, lys2/ lys2, ura3/ ura3, leu2::hisG/leu2::hisG, his3::hisG/ his3::hisG, trp1::hisG/ trp1::hisG*<br>***ECM11-9MYC**-HIS3/ECM11*	This study
LW1178	*MATa/α, ho::LYS2/ ho::LYS2, lys2/ lys2, ura3/ ura3, leu2::hisG/leu2::hisG, his3::hisG/ his3::hisG, trp1::hisG/ trp1::hisG*<br>***ECM11-9MYC**-HIS3/ECM11**; slx8::klTRP1 /slx8::klTRP1***	This study
LW1184	*MATa/α, ho::LYS2/ ho::LYS2, lys2/ lys2, ura3/ ura3, leu2::hisG/leu2::hisG, his3::hisG/ his3::hisG, trp1::hisG/ trp1::hisG*<br>***NDT80-9MYC-**HIS3/NDT80*	This study
LW1186	*MATa/α, ho::LYS2/ ho::LYS2, lys2/ lys2, ura3/ ura3, leu2::hisG/leu2::hisG, his3::hisG/ his3::hisG, trp1::hisG/ trp1::hisG*<br>***NDT80-9MYC**-HIS3/NDT80**; slx8::klTRP1 /slx8::klTRP1***	This study
LW1187	*MATa/α, ho::LYS2/ ho::LYS2, lys2/ lys2, ura3/ ura3, leu2::hisG/leu2::hisG, his3::hisG/ his3::hisG, trp1::hisG/ trp1::hisG*<br>*pHIS3-**mCherry**-**TUB1***<br>***CNM67**-**GFP-**URA3/CNM67*	This study
LW1188	*MATa/α, ho::LYS2/ ho::LYS2, lys2/ lys2, ura3/ ura3, leu2::hisG/leu2::hisG, his3::hisG/ his3::hisG, trp1::hisG/ trp1::hisG*<br>*pHIS3-**mCherry**-**TUB1***<br>***CNM67**-**GFP-**URA3/CNM67**; slx8::klTRP1 /slx8::klTRP1***	This study
LW1213	*MATa/α, ho::LYS2/ ho::LYS2, lys2/ lys2, ura3/ ura3, leu2::hisG/leu2::hisG, his3::hisG/ his3::hisG, trp1::hisG/ trp1::hisG*	This study

	FLAG-SMT3-URA3*/SMT3*	
LW1214	*MATa/α, ho::LYS2/ ho::LYS2, lys2/ lys2, ura3/ ura3, leu2::hisG/leu2::hisG, his3::hisG/ his3::hisG, trp1::hisG/ trp1::hisG*<br><br>***FLAG-SMT3****-URA3/SMT3****; slx8::klTRP1 /slx8::klTRP1***	This study
LW1215	*MATa/α, ho::LYS2/ ho::LYS2, lys2/ lys2, ura3/ ura3, leu2::hisG/leu2::hisG, his3::hisG/ his3::hisG, trp1::hisG/ trp1::hisG*<br><br>***FLAG-UBI4-SMT3****-URA3/SMT3*	This study
LW1216	*MATa/α, ho::LYS2/ ho::LYS2, lys2/ lys2, ura3/ ura3, leu2::hisG/leu2::hisG, his3::hisG/ his3::hisG, trp1::hisG/ trp1::hisG*<br><br>***FLAG-UBI4-SMT3****-URA3/SMT3****; slx8::klTRP1 /slx8::klTRP1***	This study
LW1222	*MATa/α,ho::HIS3/ ho::HIS3,arg4::KanMX4/ arg4::KanMX4, lys2::HIS3/ lys2::HIS3,GAL-NDT80::TRP1 /GAL-NDT80::TRP1*<br><br>***ECM11-AID-9MYC****-HIS3****/ECM11-AID-9MYC****-HIS3*	This study
LW1225	*MATa/α,ho::HIS3/ ho::HIS3,arg4::KanMX4/ arg4::KanMX4, lys2::HIS3/ lys2::HIS3,GAL-NDT80::TRP1 /GAL-NDT80::TRP1*<br><br>***ECM11-AID-9MYC****-HIS3****/ECM11-AID-9MYC****-HIS3****; slx8::klTRP1 /slx8::klTRP1***	This study
LW1228	*MATa/α,ho::HIS3/ ho::HIS3,arg4::KanMX4/ arg4::KanMX4, lys2::HIS3/ lys2::HIS3,GAL-NDT80::TRP1 /GAL-NDT80::TRP1*<br><br>***ZIP1-AID-9MYC****-HIS3****/ZIP1-AID-9MYC****-HIS3****; slx8::klTRP1 /slx8::klTRP1***	This study
LW1231	*MATa/α,ho::HIS3/ ho::HIS3,arg4::KanMX4/ arg4::KanMX4, lys2::HIS3/ lys2::HIS3,GAL-NDT80::TRP1 /GAL-NDT80::TRP1*<br><br>***ZIP1-AID-9MYC****-HIS3****/ZIP1-AID-9MYC-HIS3;***	This study
LW1232	*MATα/ MATa ho::LYS2/ho::LYS2 ura3/ura3 lys2/ lys2*	This study

	LEU2::ptetR-GFP<br>***HIS4::TetOx240-URA3/ HIS4::TetOx240-URA3;***	
LW1233	*MATα/ MATa ho::LYS2/ho::LYS2 ura3/ura3 lys2/ lys2*<br>***LEU2::ptetR-GFP***<br>***HIS4::TetOx240-URA3/ HIS4::TetOx240-URA3;***<br>***slx8::klTRP1 /slx8::klTRP1***	This study
LW1243	*MATa/α, ho::LYS2/ ho::LYS2, lys2/ lys2, ura3/ ura3, leu2::hisG/leu2::hisG, his3::hisG/ his3::hisG, trp1::hisG/ trp1::hisG*<br>***spo11:: KANMX4/ spo11:: KANMX4***	This study

Table S2. Plasmids Used in This Study

**Plasmid**	**Description**	**Source**
pEV0768	Ubc4 in pET28a	[2]
pHW122	*ZIP1::GFP*^{700}	[3]
pEV1068	His6-Slx5 in pET28a	This study
pEV1069	GST-Slx8 in pGEX-4t-1	This study
pEV1070	His6-FLAG-SUMO-GFP in pET21a	This study
pEV1071	His6-FLAG-SUMO2-GFP in pET21a	This study
pEV1072	His6-FLAG-SUMO4-GFP in pET21a	This study

pEV1073	His6-FLAG-SUMO4-Zip1 in pET21a	This study
pEV1074	His6-FLAG-SUMO4-Ecm11 in pET21a	This study
pEV1075	pSLX8-FLAG-Slx8 in pYC2-NT,LEU2	This study
pEV1076	pSLX8-FLAG-Slx8 RM in pYC2-NT, LEU2	This study
pEV1077	pSLX8-FLAG-Slx8 SIM*Δ* in pYC2-NT, LEU2	This study
pEV1078	pCUP1-FLAG-Smt3 in pYEP, URA3	This study
pEV1079	pCUP1-FLAG-Ubi4-Smt3 in pYEP, URA3	This study
pEV1080	His6-Slx8 in pET28a	This study
pEV1081	pSLX8-FLAG-Slx8 in pRS313, HIS3	This study
pEV1082	pSLX8-FLAG-Slx8 RM in pRS313, HIS3	This study
pEV1083	pSLX8-FLAG-Slx8 SIM*Δ* in pRS313, HIS3	This study
pNHK53	pADH1-AtTIR1-9myc	[4]
AID-9myc	pKan–AID*–9myc in pSM409	[4]

References

[1] H. Zhao, Q. Wang, C. Liu, Y. Shang, F. Wen, F. Wang, W. Liu, W. Xiao, W. Li, *Genetics* **2018**, *208,* 1181-1194.

[2] C. Liu, W. Liu, Y. Ye, W. Li, *Nat. Commun.* **2017**, *8,* 14274.

[3] H. Scherthan, H. L. Wang, C. Adelfalk, E. J. White, C. Cowan, W. Z. Cande, D. B. Kaback, *Proc. Natl. Acad. Sci. U. S. A.* **2007**, *104,* 16934-16939.

[4] M. Morawska, H. D. Ulrich, *Yeast* **2013**, *30,* 341-351.
